# Updated occurrence and bionomics of potential malaria vectors in Europe: a systematic review (2000–2021)

**DOI:** 10.1186/s13071-022-05204-y

**Published:** 2022-03-15

**Authors:** Michela Bertola, Matteo Mazzucato, Marco Pombi, Fabrizio Montarsi

**Affiliations:** 1 Istituto Zooprofilattico Sperimentale delle Venezie, Viale dell’Università 10, 35020 Legnaro, Italy; 2 Dipartimento di Sanità Pubblica e Malattie Infettive, Università di Roma “Sapienza”, P.le Aldo Moro 5, 00185 Roma, Italy

**Keywords:** *Anopheles maculipennis* s.l., *Anopheles hyrcanus* s.l., *Anopheles plumbeus*, *Anopheles superpictus*, Malaria transmission, Vector ecology, Vector behavior, Vector competence, Distribution map

## Abstract

Despite the eradication of malaria across most European countries in the 1960s and 1970s, the anopheline vectors are still present. Most of the malaria cases that have been reported in Europe up to the present time have been infections acquired in endemic areas by travelers. However, the possibility of acquiring malaria by locally infected mosquitoes has been poorly investigated in Europe, despite autochthonous malaria cases having been occasionally reported in several European countries. Here we present an update on the occurrence of potential malaria vector species in Europe. Adopting a systematic review approach, we selected 288 papers published between 2000 and 2021 for inclusion in the review based on retrieval of accurate information on the following *Anopheles* species: *An. atroparvus*, *An. hyrcanus *sensu lato (s.l.), *An. labranchiae*, *An. maculipennis *sensu stricto (s.s.), *An. messeae/daciae*, *An. sacharovi*, *An. superpictus* and *An. plumbeus*. The distribution of these potential vector species across Europe is critically reviewed in relation to areas of major presence and principal bionomic features, including vector competence to *Plasmodium*. Additional information, such as geographical details, sampling approaches and species identification methods, are also reported. We compare the information on each species extracted from the most recent studies to comparable information reported from studies published in the early 2000s, with particular reference to the role of each species in malaria transmission before eradication. The picture that emerges from this review is that potential vector species are still widespread in Europe, with the largest diversity in the Mediterranean area, Italy in particular. Despite information on their vectorial capacity being fragmentary, the information retrieved suggests a re-definition of the relative importance of potential vector species, indicating *An. hyrcanus* s.l., *An. labranchiae*, *An. plumbeus* and *An. sacharovi* as potential vectors of higher importance, while *An. messeae/daciae* and *An. maculipennis* s.s. can be considered to be moderately important species. In contrast, *An. atroparvus* and *An. superpictus* should be considered as vectors of lower importance, particularly in relation to their low anthropophily. The presence of gaps in current knowledge of vectorial systems in Europe becomes evident in this review, not only in terms of vector competence but also in the definition of sampling approaches, highlighting the need for further research to adopt the appropriate surveillance system for each species.

**Graphical Abstract:**

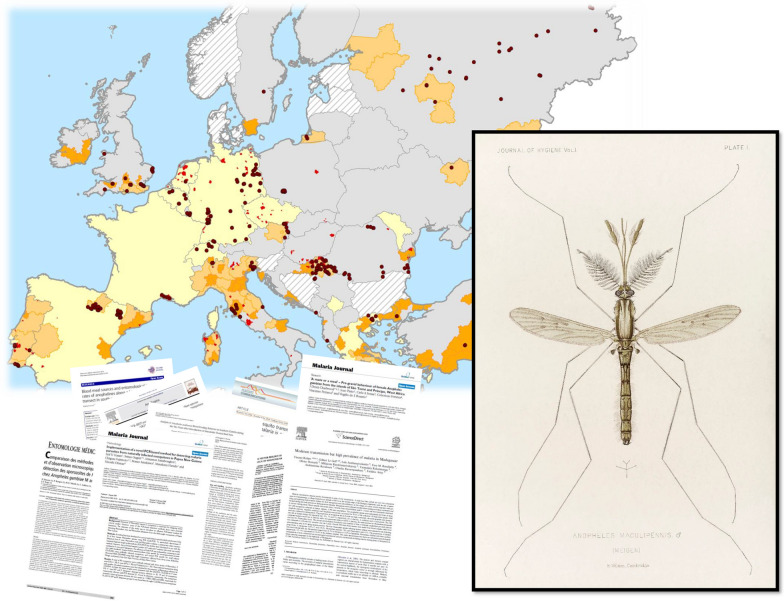

**Supplementary Information:**

The online version contains supplementary material available at 10.1186/s13071-022-05204-y.

## Background

Despite major efforts of researchers and funding institutions to tackle malaria, the most deadly of known parasitic diseases, the global burden of this life-threatening disease remains dramatic. However, great strides in combatting malaria were made in the last century, with the disease eradicated from several regions of the world, in particular in temperate areas, thanks to joint efforts at scientific, social and political levels. This disease, caused by five different species of *Plasmodium* parasites, remains a major healthcare challenge in tropical regions, particularly in sub-Saharan Africa, where *Plasmodium falciparum* and *Plasmodium vivax* are responsible for most of the morbidity.

Despite the eradication of malaria across most European countries in the 1960s and 1970s, the anopheline vectors of this disease are still present in Europe [1]. To date, most of the malaria cases reported in Europe have been infections acquired in endemic areas by travelers. However, the possibility of acquiring malaria by locally infected mosquitoes has been poorly investigated, despite autochthonous malaria cases occasionally being reported in several European countries [2].

In terms of malaria vectors in Europe, while several publications and reviews are available on the presence, density and distribution of *Anopheles* species competent for malaria transmission, most contain detailed information on countries where malaria is endemic [3–5]. To help researchers study malaria transmission, maps of the global distribution of malaria vector species have been created [6–9], most of which focus on tropical endemic areas.

Several potential *Anopheles* malaria vectors have been identified at various locations throughout Europe, but data on these species are less structured and often collected within the framework of monitoring plans focused on other mosquito species. Precise data on the presence of *Anopheles* mosquitoes is limited, with complete information available only for some European countries.

A comprehensive picture of *Anopheles* distribution at the European level was provided in three reviews [10–12]. Subsequent to the publication of these reviews, the last study that reviewed the occurrence and geographic distribution of the dominant vector species of human malaria (DVS; with “dominant” defining a vector species that has been identified as the main, dominant or important vector in at least one region) was published in 2010 by Sinka et al. [4] within the framework of the Malaria Atlas Project (MAP) [13]. Recent publications, mostly driven by the use of the more novel molecular diagnostic techniques applied in recent years [14], provide the basis for a novel critical review of the occurrence of *Anopheles* species, in particular a review of those species not included in the category of DVS but potentially involved in malaria transmission that have not previously been considered.

We present here an updated systematic review on the occurrence of potential mosquito malaria vectors in Europe, with a specific focus on studies published in the last 20 years, with the aim to provide a critical revision of the species that should be considered of major importance for local malaria transmission. The accurate selection of peer-reviewed literature available since 2000 has allowed the retrieval of information on the occurrence of *Anopheles* target species in European countries, highlighting the areas of major interest, and critical descriptions of major aspects of species ecology, behavior and vector competence. The most important *Anopheles* species potentially involved in local transmission events are critically reviewed in terms of their epidemiological relevance and presence in Europe.

## Methods

**Fig. 1:**
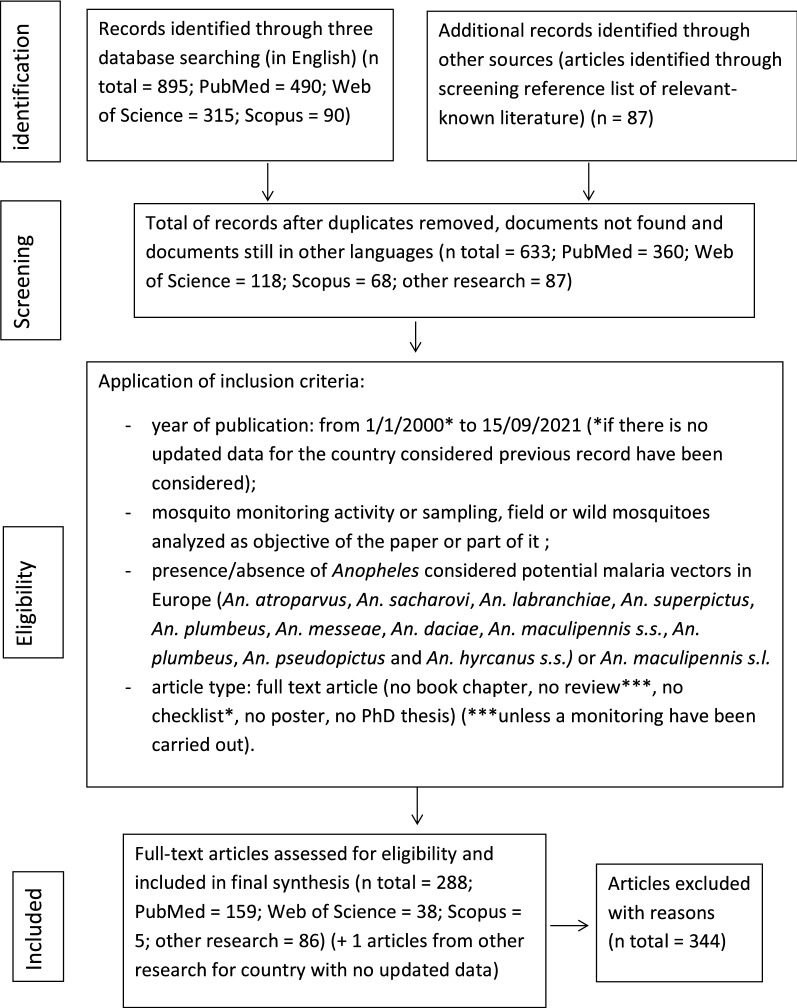
PRISMA flow diagram of eligible study selection process

The information retrieved from published literature for this review was collected following the reporting guidelines of the Preferred Reporting Items for Systematic Reviews and Meta-Analyses (PRISMA) Statement for systematic reviews and Meta-Analyses [15] (Fig. 1).

### Eligibility criteria of literature search

Across the world, 41 mosquito species (and species complexes) are considered to be “main” or “potential” human malaria vectors [16]. Based on two major reviews [4, 16], the DVS considered in this review are *Anopheles atroparvus*, *Anopheles sacharovi*, *Anopheles labranchiae*, *Anopheles messeae/daciae* and *Anopheles superpictus*. Additionally, we included *Anopheles plumbeus* and European species belonging to the Hyrcanus Group (hereafter referred to as *Anopheles hyrcanus* sensu lato [s.l.]) in this review based on the findings of recent studies indicating their potential role as malaria vectors in some geographic areas. Lastly, *Anopheles maculipennis *sensu stricto (s.s.) is also taken into consideration; although this species is considered to be a secondary malaria vector, its wide distribution and local abundance make it a potential malaria vector in some areas. The criteria of selection of species are based on their occurrence, abundance, biology, evidence or suspicion of involvement in malaria transmission (also at a local level) and vectorial competence. To retrieve a more complete set of data, the taxonomic definition *An. maculipennis *s.l. was also searched. Some authors formally state that *An. daciae* is a new species [17], while others consider it to be a genetic polymorphism within *An. messeae* taxon [18]. Taking into account that this question is still being debated and that data on each taxon are still scarce and difficult to unequivocally address, we did not separate these two taxa in this review, and refer to them as *An. messeae/daciae.* In addition, *An. hyrcanus* is a taxonomic group of closely related *Anopheles* species that are difficult to distinguish, some of which are considered to be malaria vectors. Due to the uncertainty in the description of these species in the literature, we refer to them here as *An. hyrcanus* s.l. [19, 20].

In this review we focus on malaria vectors in Europe, but we also include additional countries based on a compromise between the geographical and political borders of Europe. Therefore, we have included Turkey, Azerbaijan, Georgia and Armenia, as well as European Russia, in this review, but excluded Kazakhstan. Data on Cyprus have been collected referring to the whole island (not considering political boundaries). Islands not located in the Mediterranean Sea and overseas territories are excluded. Countries with a limited surface, such as Lichtenstein, Andorra, San Marino, Vatican City State and the Principality of Monaco were also not included, since no data are available for any of these.

Due to the heterogeneity in sampling methods and geographic details of occurrence available in the retrieved literature, data on mosquito abundance are not taken into account, and only presence/absence data are included.

In order to represent the most current possible representation of *Anopheles* vector presence, only studies published between 2000 and 2021 were considered. However, articles published within this time frame but reporting samplings made prior to the study period were included in the review. Articles published before 2000, if available, were taken into account only for countries for which more recent literature was not available.

Only full-text articles providing original research and data were screened; book chapters, conference abstracts, checklists, posters, PhD theses, among others (gray literature) were excluded. Reviews were also excluded, unless original data were reported.

### Information sources

Three online databases (PubMed [21], Web of Science [22] and Scopus [23]) were searched for relevant scientific literature. Additionally, cited references cited in the retrieved articles were checked (other research).

### Search strategy

The systematic research and review were performed on studies published from January 2000 to September 2021. The search was conducted in English using the following keywords: “*Anopheles*” and “*messeae*” or “*maculipennis*” or “*daciae*” or “*messeae/daciae*” or “*atroparvus*” or “*labranchiae*” or “*sacharovi*” or “*superpictus*” or “*pseudopictus*” or “*hyrcanus*” or “*plumbeus*”, in association with any one of the 45 countries considered: Albania, Armenia, Austria, Azerbaijan, Belarus, Belgium, Bosnia-Herzegovina, Bulgaria, Croatia, Cyprus, Czech Republic, Denmark, Estonia, Finland, France, Georgia, Germany, Greece, Hungary, Iceland, Ireland, Italy, Kosovo, Latvia, Lithuania, Luxembourg, Macedonia, Malta, Moldova, Montenegro, Netherlands, Norway, Poland, Portugal, Romania, Russia, Serbia, Slovakia, Slovenia, Spain, Sweden, Switzerland, Turkey, Ukraine, UK.

### Study selection

As the first step, one of the authors of this review performed the literature search, removing duplicates (same articles obtained more than once during different searches), full-text documents not published in English and separating the retrieved articles into two groups according to the year of publication (before or after 2000). As the second step, the same author, who had read all the retrieved articles, performed the eligibility assessment; only those articles which met the above-mentioned eligibility criteria were selected and used in this review. Then, a second author checked and repeated the process to evaluate agreement within the papers screened by the first author. Disagreements between authors were resolved by a third author. At the end of the study selection process, a final list of selected articles was obtained.

### Data collection process

Following the eligibility assessment, a database reporting the following information from each selected article was created: bibliographical details (title, authors, journal, year of publication, DOI and database source), European countries considered, species occurrence, monitoring/field activity, year of monitoring activity, identification method(s) and sampling technique(s) (specifying if outdoor or indoor) (see Additional file 1).

In particular, adult mosquito capture methods were classified as: (i) active manual (aspiration, use of nets); (ii) mechanical visual (traps using visual attractants such as light, colors, shapes); (iii) mechanical olfactory (traps using olfactory attractants, such as CO_2_, olfactory lures or water); (iv) human-baited (human landing catches or human-baited traps); (v) animal-baited (animal-baited traps). Only one capture method for larval collection is defined, namely dipping/hand collection; larvae or eggs collected by ovitraps are also specified separately.

Information on the ecology and behavior of the potential malaria vectors was also extracted for each species. In particular, breeding sites and feeding behavior were summarized in categories and displayed in tables. This review does not include any information relating to insecticide resistance since it is a topic not well addressed for the European *Anopheles* species.

After the selection process, two authors separately read all of the articles and checked whether all of the information had been correctly extracted. The other two authors double-checked and verified the accuracy of the information reported. Any disagreements between reviewers were resolved.

### Geographical analysis

**Fig. 2:**
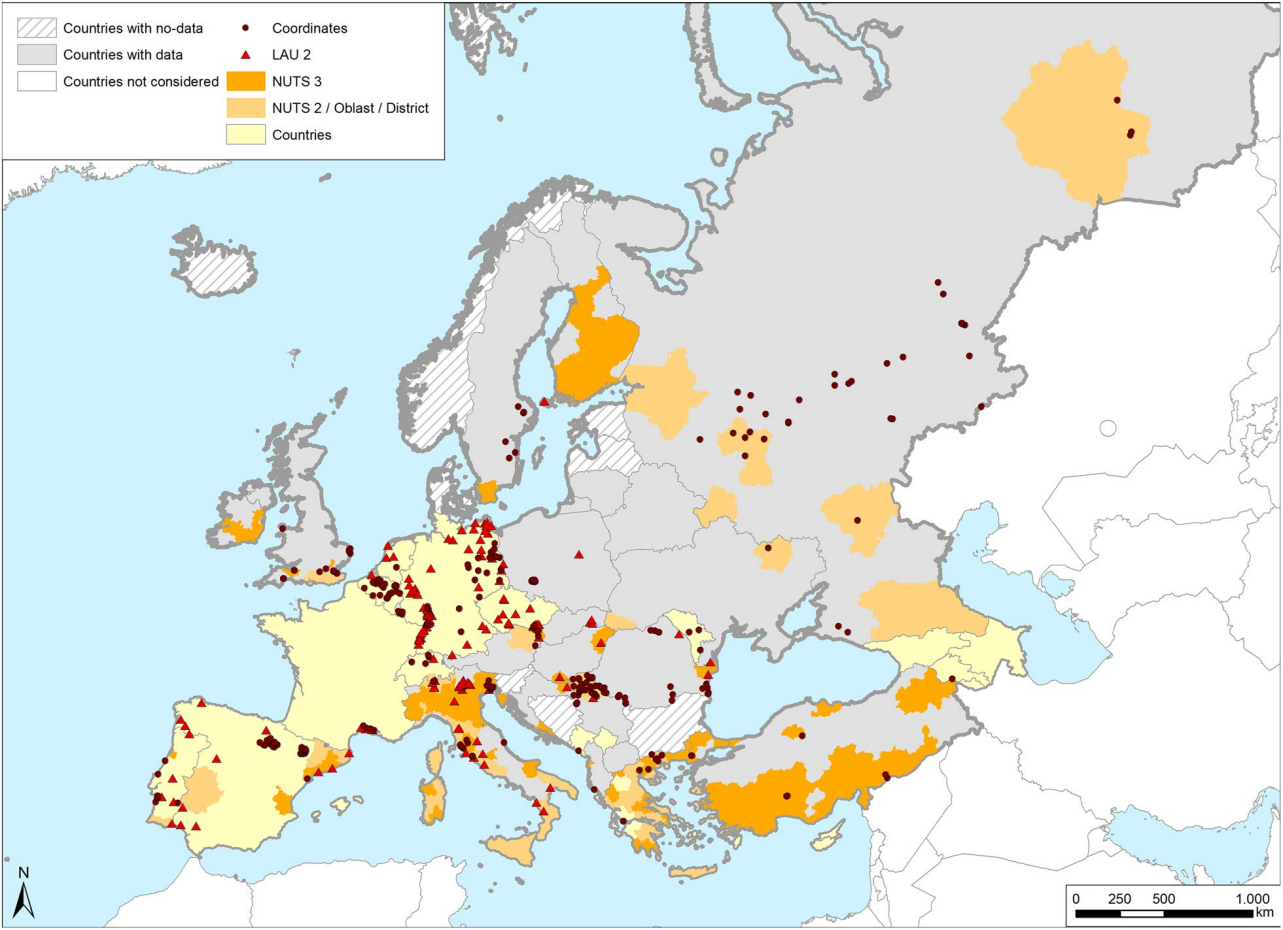
Occurrence map of potential malaria vector species in Europe at different geographical levels (see legend at upper-left of figure) according to retrieved literature (January 2000–September 2021).* Abbreviations*: LAU, Local Administrative Units; NUTS 1, 2 3, Nomenclature of Territorial Units for Statistics levels 1, 2, 3, respectively; Oblast, alternative Nominal code for Russia and Ukraine

**Fig. 3:**
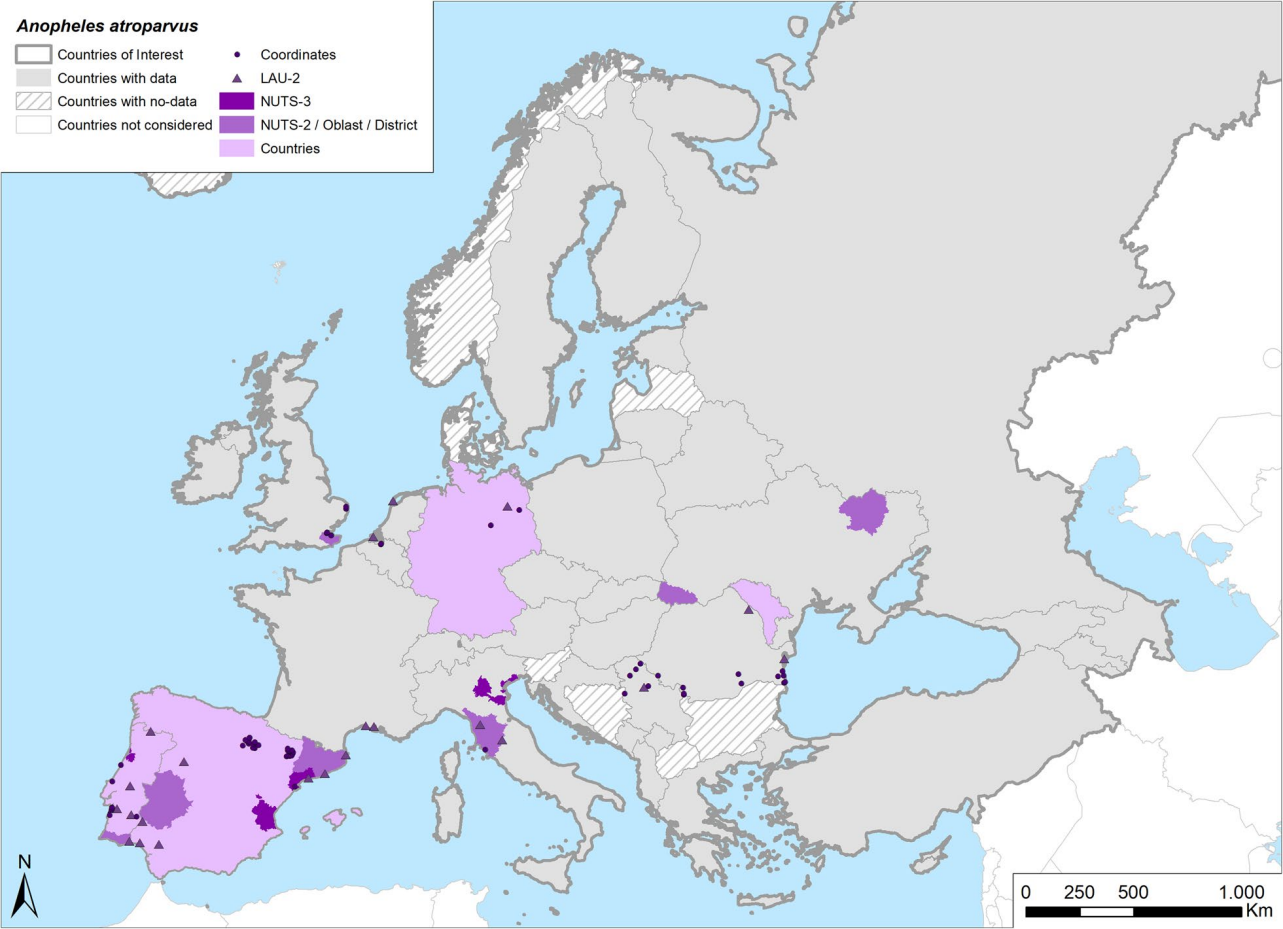
Occurrence map of *Anopheles atroparvus* in Europe at different geographical levels (see legend at upper-left of figure) according to retrieved literature (January 2000–September 2021)

**Fig. 4:**
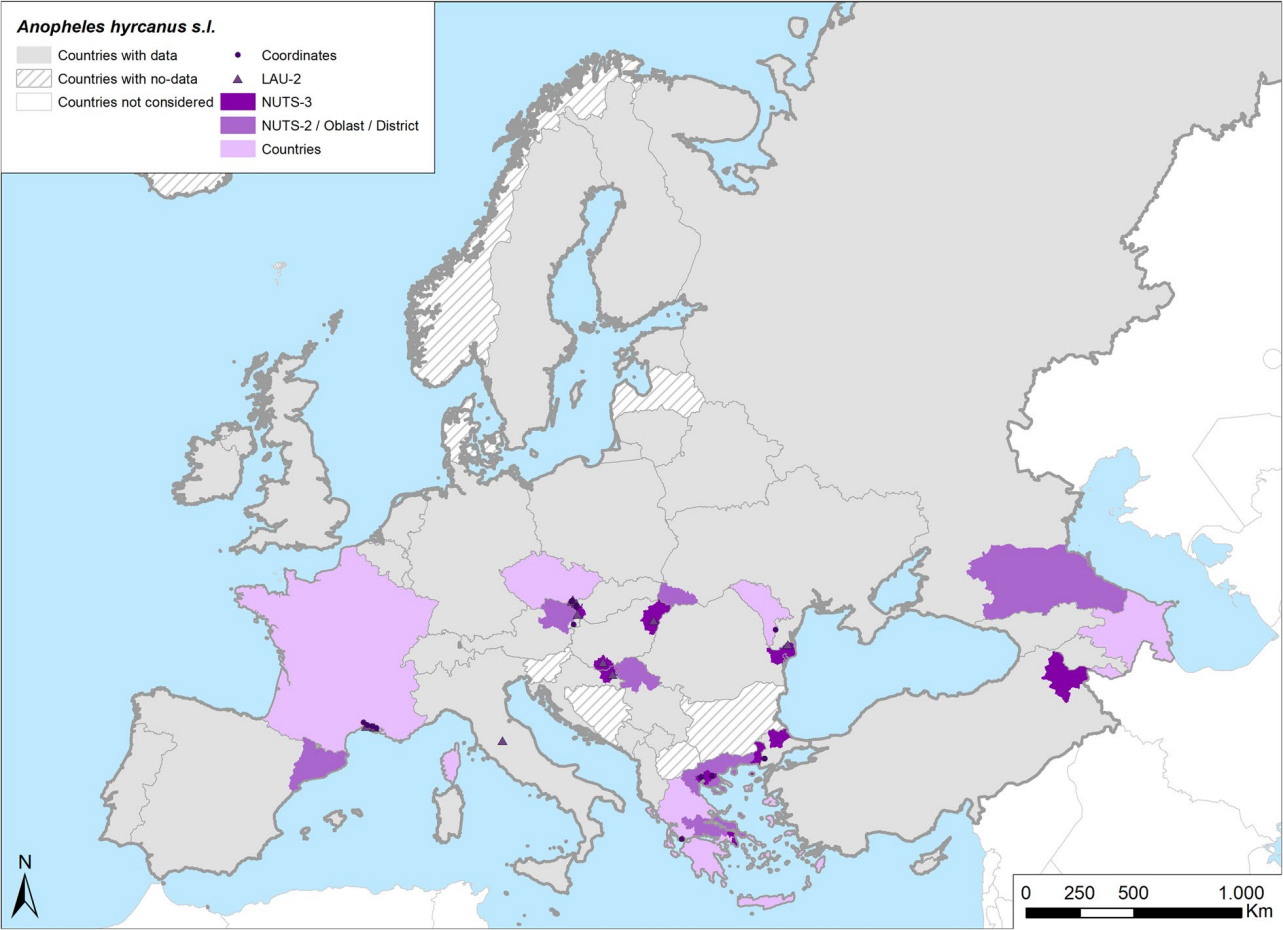
Occurrence map of *Anopheles hyrcanus* s.l. in Europe at different geographical levels (see legend at upper-left of figure) according to retrieved literature (January 2000–September 2021)

**Fig. 5:**
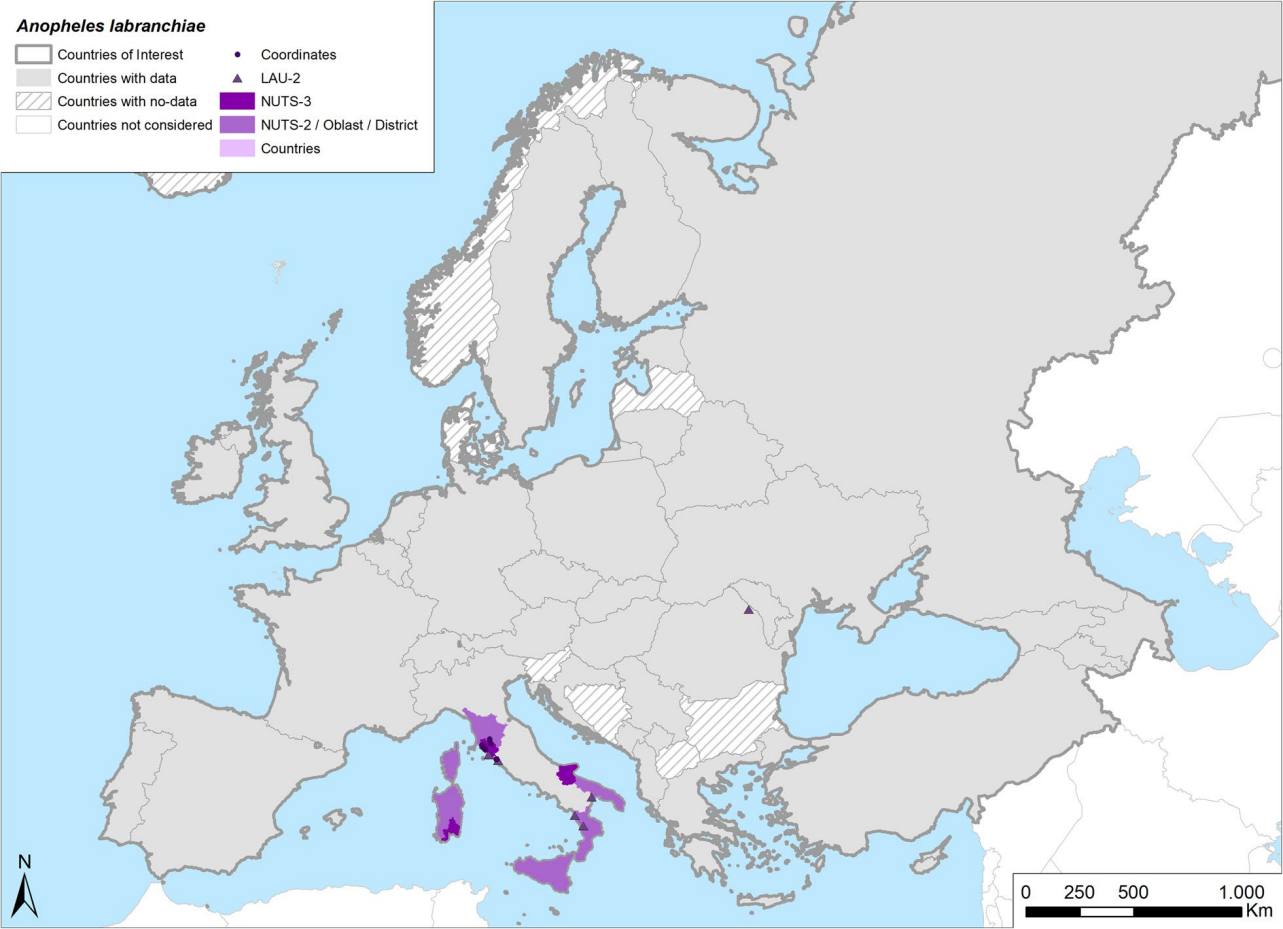
Occurrence map of *Anopheles labranchiae* in Europe at different geographical levels (see legend at upper-left of figure) according to retrieved literature (January 2000–September 2021)

**Fig. 6:**
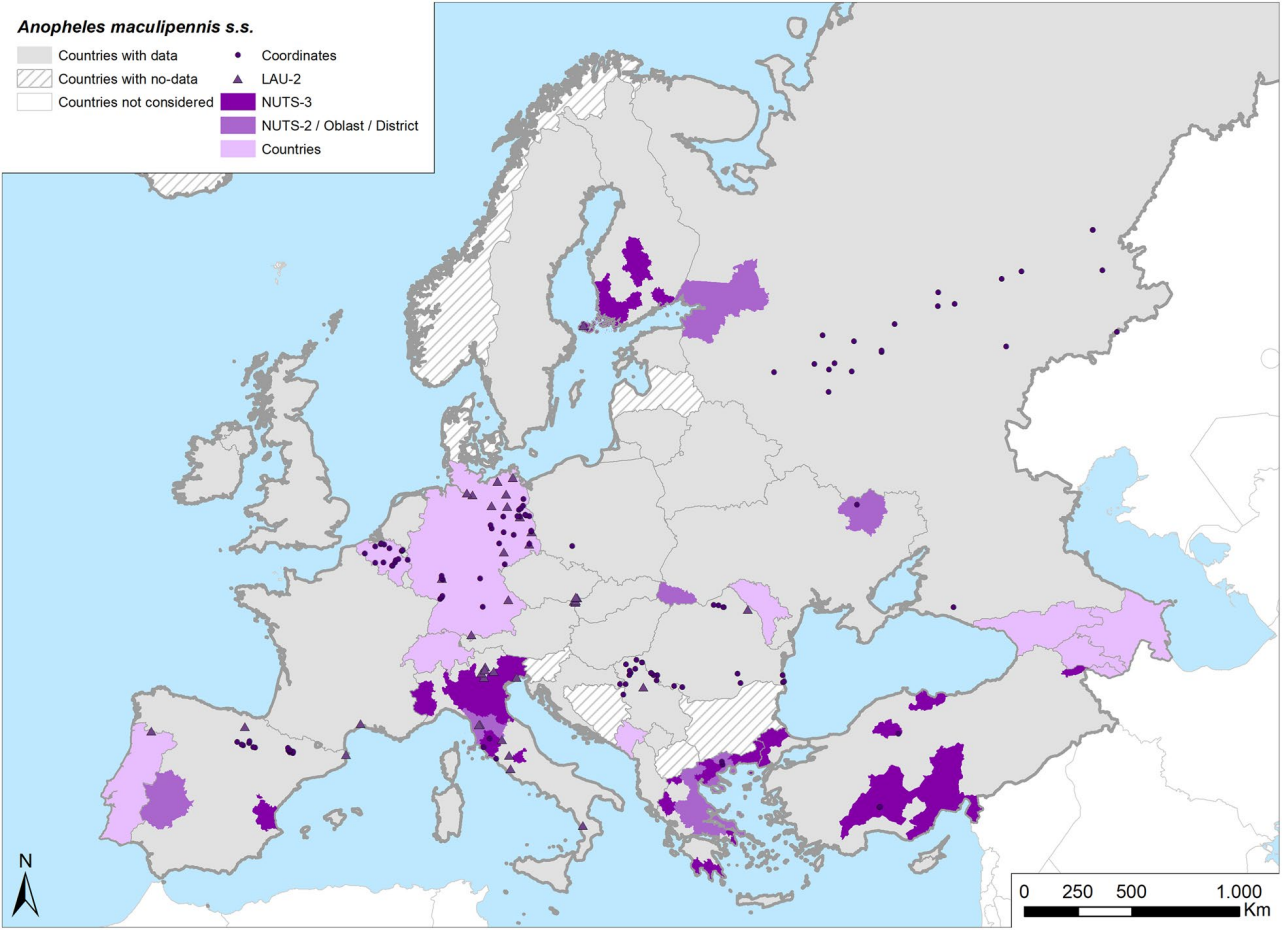
Occurrence map of *Anopheles maculipennis* s.s. in Europe at different geographical levels (see legend at upper-left of figure) according to retrieved literature (January 2000–September 2021)

**Fig. 7:**
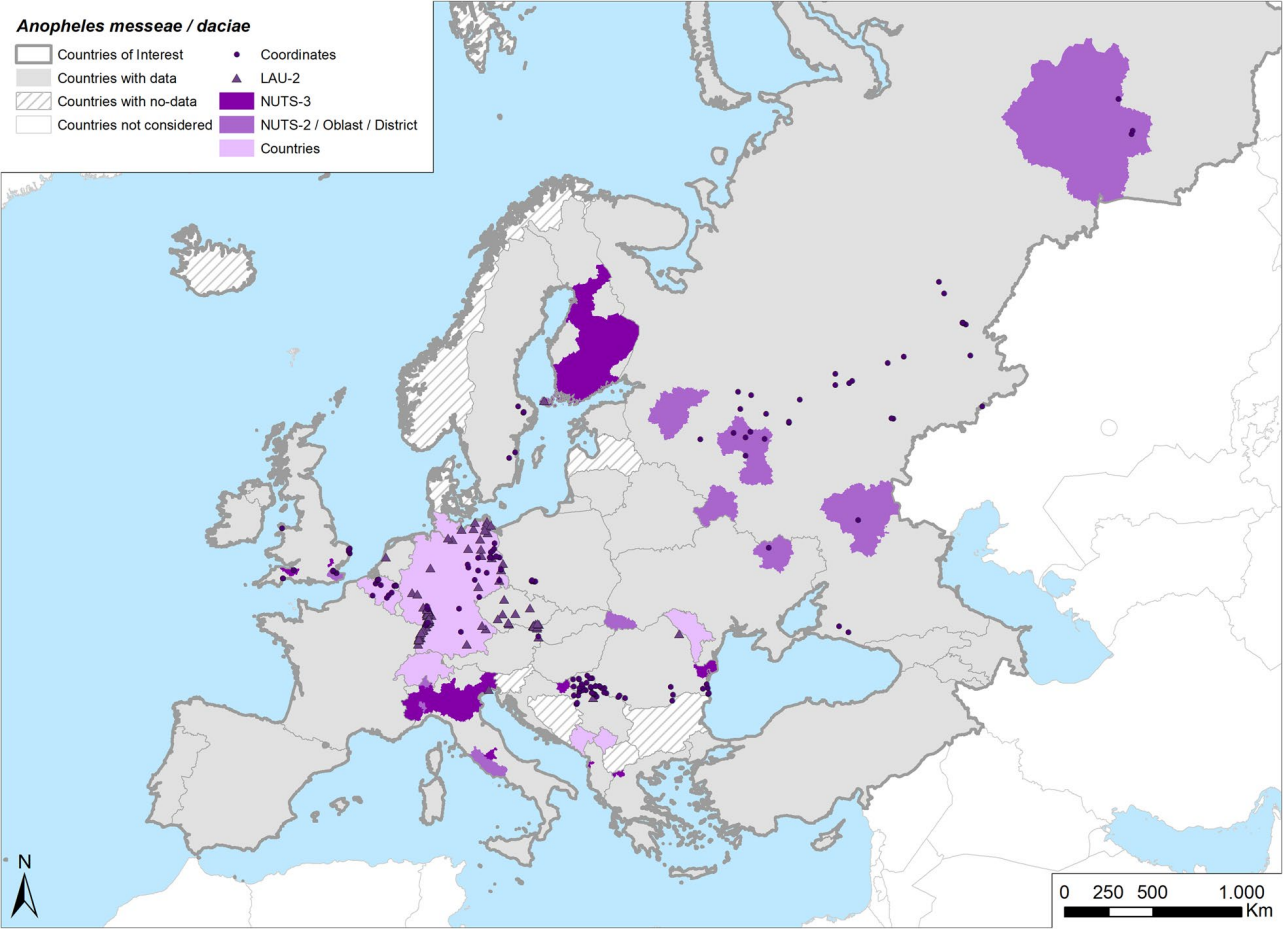
Occurrence map of *Anopheles messeae/daciae* in Europe at different geographical levels (see legend at upper-left of figure) according to retrieved literature (January 2000–September 2021)

**Fig. 8:**
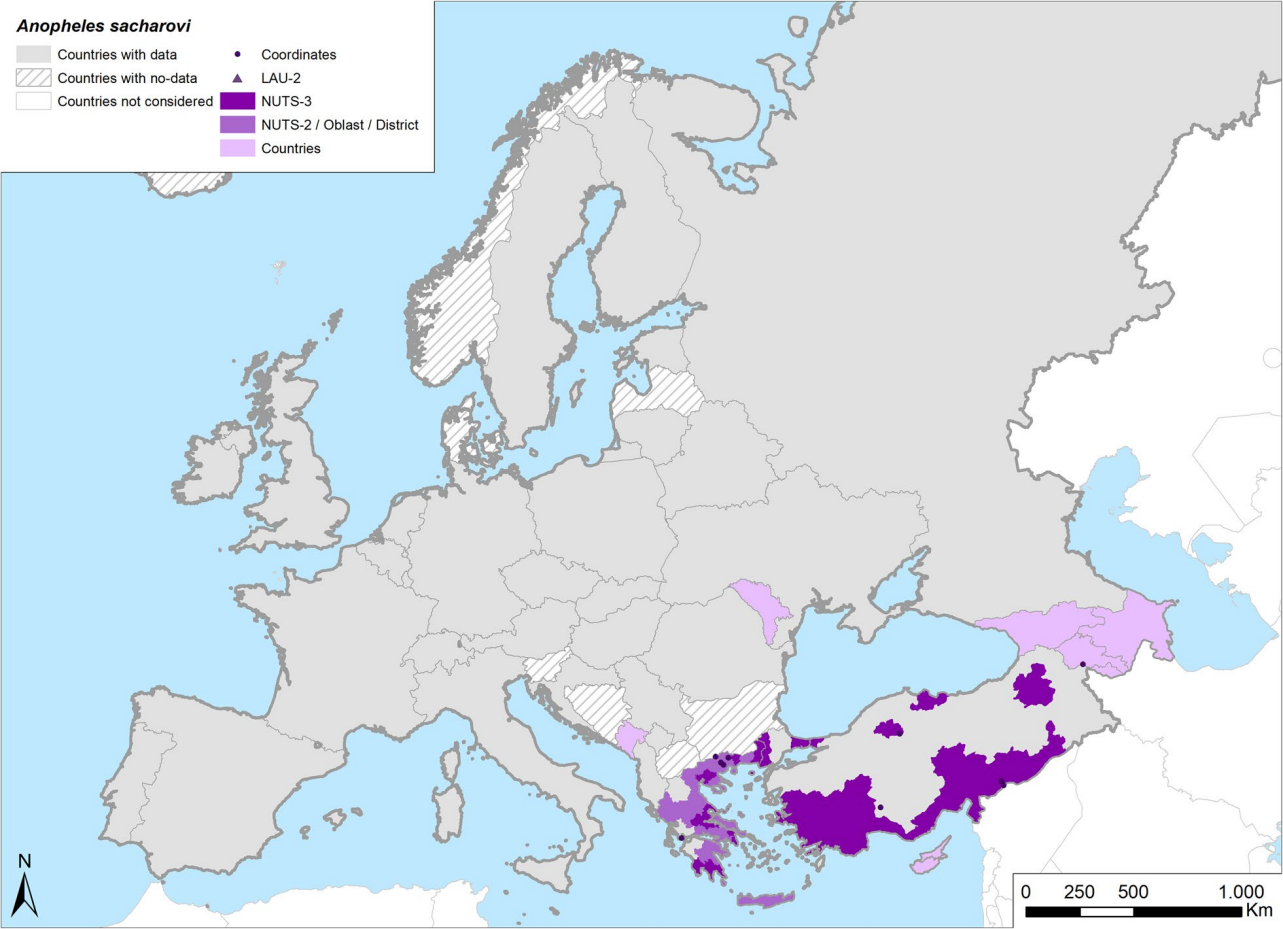
Occurrence map of *Anopheles sacharovi* in Europe at different geographical levels (see legend at upper-left of figure) according to retrieved literature (January 2000–September 2021)

**Fig. 9:**
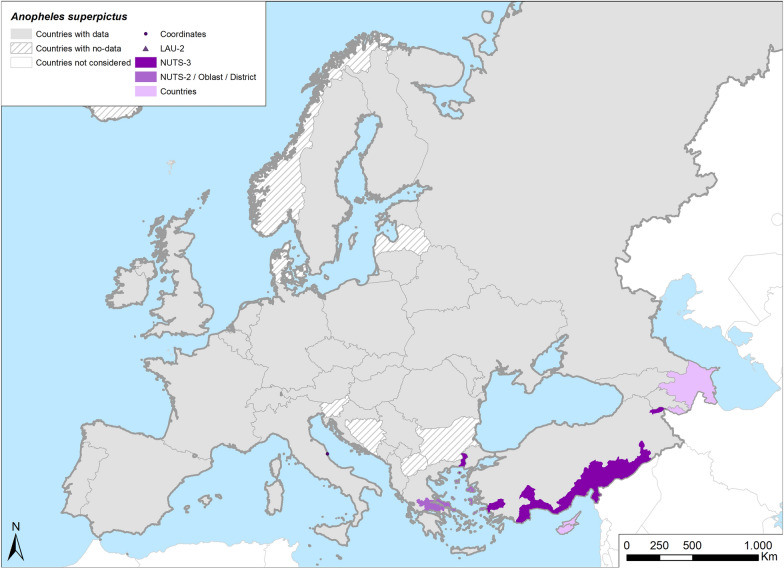
Occurrence map of *Anopheles superpictus* in Europe at different geographical levels (see legend at upper-left of figure) according to retrieved literature (January 2000–September 2021)

**Fig. 10:**
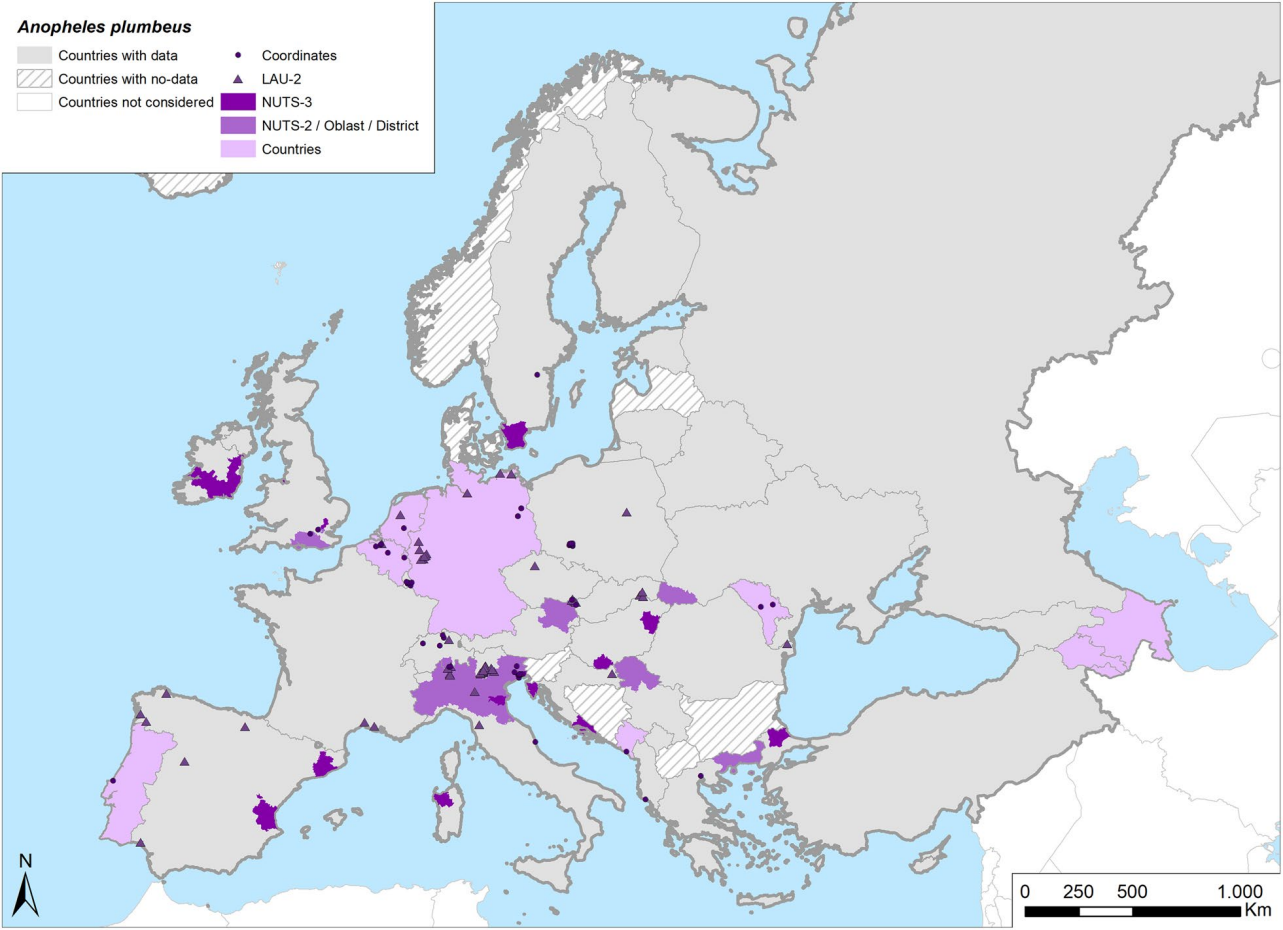
Occurrence map of *Anopheles plumbeus* in Europe at different geographical levels (see legend at upper-left of figure) according to retrieved literature (January 2000–September 2021)

Geo-referenced data reported in the maps (Figs. 2–10) were classified into the three categories according to their availability: (i) GPS coordinates (of each collection site); (ii) area-zone (when multiple sites were sampled in a specific area or the name of this area was mentioned); (iii) country (when the geographic information was not specified for the country sampled).

Data on the area/zone of EU member countries, candidate countries and members of the European Free Trade Association are classified according to the Nomenclature of Territorial Units for Statistics (NUTS), which has three levels (NUT1, NUTS2, NUTS3) [24]. This classification establishes a correspondence between the NUTS levels and the national administrative units. Local Administrative Units (LAU) were also used when it was possible to trace the municipality of the collection and circumscribe a more precise area within NUTS3 level. In cases where the areas involved different territorial units, they have been split into single territorial units to better show data in maps.

For countries with no NUTS classification (Armenia, Azerbaijan, Kosovo, Moldova, Montenegro and Cyprus), the widest geographic level was conservatively adopted (country level). For Russia and Ukraine, the Nominal Code named Oblast has been used as an alternative to NUTS classification. It was not possible to find the Nominal Code of the Republic of Dagestan (Russia); therefore, we enlarged the area to the North Caucasus Federal District and that Nominal Code was used.

When the presence of an *Anopheles* species has not been assigned to a specific site, all of the sites monitored were considered to be negative for that species (marked as Not Assigned “NA” Additional file 1), and the species occurrence reported at an upper geographical level.

Based on the above-mentioned classification, we created a European occurrence map for all potential malaria vector species (Fig. 2) and for each species separately (Figs. 3–10) based on the retrieved literature.

Maps and spatial data manipulation were carried out using ESRI ArcMap (ArcGIS Desktop: Release 10.5.1; Environmental Systems Research Institute, Redlands, CA, USA; copyright 1999–2017).

### Data items and risk of bias in individual studies

Articles with unclear or questionable information were excluded; for example, articles on the occurrence of potential malaria vectors not supported by data. Only articles containing original data are recorded in the database. Therefore, articles reporting modeling based on data obtained from other studies were not included.

## Summary of extracted data

The search of the three databases resulted in the identification of 982 publications (Fig. 1), 490 in PubMed, 315 in Web of Science and 90 in Scopus, with subsequent review of the citations from retrieved articles identifying a further 87 studies. Of these, 633 papers (531 published after 2000 and 102 published before 2000) remained after the removal of duplicate studies and articles not published in English.

Of the 531 articles published after 2000, 288 fulfilled the inclusion criteria; the other publications were discarded due to: (i) no potential malaria vectors found in the monitoring activity of the considered country; (ii) no original data presented in the study; (iii) studies focused on other topics and/or presentation of data/monitoring activity was unclear. Of the 102 full-text articles published before 2000, only one fulfilled the inclusion criteria; for the other 101 articles, more recent data were available for the country considered.

The occurrence of potential malaria vectors in European countries is shown in Fig. 2 and summarized in Table 1; in 33 of the 45 screened European countries, at least one potential malaria vector was reported. The largest amount of data came from Italy (32 references), which is the only country reporting the presence of all of the *Anopheles* species investigated in this review. In contrast, only one or two studies were reported for 12 countries, in particular from countries in the Eastern part of Europe. Data from Belarus, Estonia and Lithuania reported the presence of specimens belonging to the *An. maculipennis* complex. No data were found for Bosnia-Herzegovina, Bulgaria, Denmark, Iceland, Latvia, Macedonia, Malta, Norway and Slovenia.

**Table 1:** References reporting the occurrence of potential malaria vectors in European countries

Country	Potential malaria vectors in European countries^a^									
	*atr*	*hyr* s.l.	*lab*	*mac* s.s.	*mes/dac*	*plu*	*sac*	*sup*	*mac* s.l.	References
Albania					ML	MF			MF	[88, 170]
Armenia				ML			ML		MF	[28, 88, 112, 139, 150, 171]
Austria		MF				MF			MF	[74, 86, 172–176]
Azerbaijan		MF					ML	MF	MF	[88, 139, 177]
Belarus									MF	[178]
Belgium				ML	ML	MF			MF	[117, 143–145, 179–186]
Croatia		MF				MF			MF	[146, 187–193]
Cyprus							MF	MF		[141, 142]
Czech Republic		MF		ML	ML	MF			MF	[37–39, 79, 80, 155, 194–203]
Estonia				ML			ML			[204]
Finland				ML	ML				MF	[100–102]
France	ML	ML	ML	ML		MF			MF	[20, 30, 33, 34, 68–70, 77, 78, 87, 88, 203, 205–207]
Georgia										[139]
Germany	ML			ML	ML	MF			MF	[41–44, 111, 119–122, 151, 152, 159, 163, 208–219]
Greece		ML		ML	ML	MF	ML	MF	MF	[103, 104, 107, 110, 130, 135, 136, 203, 220–231]
Hungary		MF							MF	[82, 170, 232–234]
Ireland						MF			MF	[235]
Italy	ML	MF	ML	ML	ML	MF	MF		MF	[28–32, 52, 60, 88–91, 93, 94, 97, 99, 128, 138, 236–250]
Kosovo					ML				MF	[251]
Lithuania									MF	[252]
Luxembourg						MF			MF	[253]
Moldova	ML	MF		ML	ML	MF	ML		MF	[81, 254, 255]
Montenegro				ML	ML	MF	MF		MF	[170, 256]
The Netherlands	ML				ML	MF			MF	[35, 45, 88, 128, 154, 257, 258]
Poland				ML	ML	MF			MF	[124, 259–263]
Portugal	ML			ML		MF			MF	[25–27, 30, 46, 57, 264–271]
Romania	ML	MF	ML	ML	ML	MF			MF	[17, 30, 92, 272–274]
Russia		MF		ML	ML				MF	[18, 28, 75, 98, 115, 118, 125, 275–283]
Serbia	ML	MF		ML	ML	MF			MF	[108, 113, 284–287]
Slovakia	ML	MF			ML	MF			MF	[39, 40, 288–293]
Spain	ML	MF		ML		MF			MF	[30, 36, 47–51, 59, 61, 149, 165, 294–306]
Sweden						MF			MF	[116, 128, 307–310]
Switzerland				ML	ML	MF			MF	[148, 158, 245, 311–315]
Turkey		ML		ML		MF	ML	MF	MF	[20, 28, 71–73, 88, 105, 106, 109, 131–133, 140, 316–327]
Ukraine	MF			MF	MF				MF	[328, 329]
UK	ML				ML	MF			MF	[53–56, 58, 123, 126, 127, 147, 330–336]

MF, morphological identification; ML, molecular identification

^a^*atr*, *Anopheles atroparvus*; *hyr* s.l., *Anopheles hyrcanus* sensu lato; *lab*, *Anopheles labranchiae*; *mac* s.s., *Anopheles maculipennis* sensu stricto; *mes/dac*, *Anopheles messeae/daciae*; *plu*, *Anopheles plumbeus*; *sac*, *Anopheles sacharovi*; *sup*, *Anopheles superpictus*; *mac* s.l., *Anopheles. maculipennis* sensu lato

*Anopheles plumbeus* was the most recorded species and was found in 29 European countries; however, this species was generally reported in low numbers, within the framework of entomological surveys focused on other mosquito species. Among the other targeted *Anopheles*, *An. messeae/daciae* and *An. maculipennis* s.s. are widespread in Europe (found in 21 and 22 countries, respectively), with the former particularly present in central and northern Europe. Findings on *Anopheles hyrcanus* s.l. and *An. atroparvus* are scattered across Europe (detected in 16 and 12 countries, respectively). As already reported in the literature, the presence of *An. sacharovi* and *An. superpictus* in south-eastern Europe was confirmed; similarly, the least prevalent of the mosquito species studied here, *An. labranchiae*, was reported only in Romania, Italy,and Corsica (France) (Table 1).

Specifically, *Anopheles* target species were found in 54.9% of the 1925 geographic records retrieved in the systematic review. *Anopheles messeae/daciae* was the most reported of these(*n* = 383, 36.3%), followed by *An. maculipennis* s.s. (*n* = 365, 34.6%) and *An. sacharovi* (*n* = 204, 19.3%). The least prevalent species were *An. superpictus* and *An. labranchiae* (*n* = 33, 3.1% and *n* = 27, 2.6%, respectively) (Additional file 1).

Information retrieved on the collected samples indicated that adult mosquito catches was performed in 161 studies, larval/pupal collections in 35 studies and a combination of egg, larvae and adult collections were performed in 93 studies (Additional file 1).

Sampling methods were described in 278 of 288 papers, and the most common sampling method reported among all studies was adult mosquito catches using traps baited with CO_2_, octenol or other lures (mechanical olfactory method; 115/254, 45.3% of studies). Only 28.8% of the 278 articles selected for inclusion in this review reported samplings planned specifically for *Anopheles* collection; the others referred to monitoring activities for detection of other mosquito species or surveillance planning for arboviral diseases. Considering only those papers which specifically aimed at human malaria vector surveillance and/or collection, the most used method to collect *Anopheles* species was manual collection (40/80, 50%) (Additional file 1).

Molecular identification was reported in 94 articles (32.5% of the total), with the primary aim to identify the species belonging to the *An. maculipennis* complex. Molecular diagnostics were also used to identify *An. plumbeus* and *An. hyrcanus* s.l. (2 and 4 records, respectively). In terms of the technique/method applied, the use of PCR methodology targeting the internal transcribed spacer 2 (ITS2) of ribosomal DNA was the most used approach in our dataset and was applied in 81/94 studies. Less commonly used methods included assays on other gene targets, such as fragments of the mitochondrial cytochrome oxidase I (*cox1*) gene (23 records),* 28S* ribosomal DNA (3 records) or other genes.

## Updates on the occurrence and bionomics of the potential vector species

Based on the information retrieved from systematic review of the three databases, in the following sections we provide an update of the occurrence and bionomics of each potential malaria vector species investigated in this review. For each species, we make a critical comparison: the data acquired from recent references screened in this review are summarized (Tables 2 and 3) and compared with information reported in the literature before 2000.

**Table 2:** Breeding sites of potential malaria vector species

Mosquito species	Total number of reported breeding sites	Lagoons, brackish waters	Marshes, swamps, ponds, overflow rivers	Puddles, pools, pits	Irrigation channels	Rice fields	Artificial containers
*An. atroparvus*	32	6 (18.7)	6 (18.7)	4 (12.5)	7 (21.9)	8 (25.0)	1 (3.1)
*An. hyrcanus s.l*	17		12 (70.6)			5 (29.4)	
*An. labranchiae*	12	1 (8.3)	2 (16.7)	2 (16.7)	4 (33.3)	3 (25.0)	
*An. maculipennis* s.s.	31		11 (35.5)	6 (19.3)	9 (29.0)	4 (12.9)	1 (3.3)
*An. messeae/daciae*	24		12 (50.0)	4 (16.7)	5 (20.8)	2 (8.3)	1 (4.2)
*An. plumbeus*	23		1 (4.3)	7 (30.4)			15 (65.2)
*An. sacharovi*	19	2 (10.5)	8 (42.1)	1 (5.3)	6 (31.6)	2 (10.5)	
*An. superpictus*	4		2 (50.0)		2 (50.0)		

Data presented in table are the number (and percentage) of types of reported breeding sites as reported in the retrieved literature (January 2000–September 2021)

**Table 3:** Feeding behavior of potential malaria vector species

Species	Total number of reports	Human	Equid	Cattle	Small ruminants (sheep, goat)	Pig	Dog	Rabbit	Chicken	Birds other than chicken	Other mammals
*An. atroparvus*	26	3 (11.5)	4 (15.4)	4 (15.4)	2 (7.7)		3 (11.5)	3 (11.5)	2 (7.7)	3 (11.5)	2 (7.7)
*An. hyrcanus* s.l.	12	8 (67)	2 (17)							2 (17)	
*An. labranchiae*	11	4 (36.4)	2 (18.2)	3 (27.2)		2 (18.2)					
*An. maculipennis* s.s.	14	3 (21.4)	2 (14.3)	2 (14.3)	3 (21.4)	1 (7.1)			1 (7.1)	1 (7.1)	1 (7.1)
*An. messeae/daciae*	37	11 (29.7)	4 (10.8)	9 (24.3)	4 (10.8)		4 (10.8)		2 (5.4)	1 (2.7)	2 (5.4)
*An. plumbeus*	13	8 (61.5)			1 (7.7)				1 (7.7)	2 (15.4)	1 (7.7)
*An. sacharovi*	2			1 (50.0)	1 (50.0)						
*An. superpictus*	0										

Data presented in table are the number (and percentage) of reports of host species as reported in the retrieved literature (January 2000–September 2021)

### *Anopheles atroparvus* Van Thiel 1972

#### Occurrence

*Anopheles atroparvus* is widely distributed in Europe, with a distribution ranging from Portugal to the UK and Ukraine (Table 1; Fig. 3). Based on the data presented in this systematic review, this potential malaria vector is still abundant with medium–high densities in the Iberian Peninsula (for which it was historically considered the main malaria vector) and along the coastal region of northern Europe, in confirmation with older literature records [10, 11]. In Portugal, *An. atroparvus* is still reported throughout the country but particularly in the south and central areas [25, 26], with a spatial distribution pattern overlapping previously recorded malarial transmission areas [27]. In contrast, its occurrence is now sporadic in Mediterranean regions: in Italy, it was recorded in scattered sites of northern and central Italy [28–32]; in France, its occurrence is reduced as compared to the 1940s and 1950s [33, 34]. It has been suggested that one or more factors may have affected its current presence, such as changing land use, pollution, use of control methods (e.g. insecticides or predatory fishes in rice fields), lack of suitable larval habitats and feeding and resting sites [35, 36]. *Anopheles atroparvus* also shows a marked decrease in occurrence in central European countries: it has not been recorded since 2000 in the Czech Republic [37, 38] and Slovakia [39, 40], but is still present in Germany [41–44].

#### Breeding sites

*Anopheles atroparvus* has been traditionally described as a species more tolerant to salinity as compared to the other species of the complex (*An. maculipennis* s.l.), with a preference for brackish water breeding sites. However, it has also been reported in freshwater larval habitats, such as temporary pools, puddles, irrigation channels, river margins and rice fields. The retrieved literature contained reports of this species still being found in coastal areas, confirming the link with habitats characterized by a certain degree of salinity, in particular along the coast of Portugal, the Netherlands and Germany [27, 43–46]. The current occurrence of *An. atroparvus* seems also to be related to the presence of water supplies for agricultural purposes, which is particularly abundant in rice-growing areas; according to the retrieved data, in the absence of mosquito control activities rice crops were strongly colonized by this species [26, 46–48]. Irrigation channels are the breeding sites where *An. atroparvus* larvae were most frequently reported, followed by rice fields and large water basins (e.g. lagoons, marshes, swamps), both permanent and temporary, containing brackish or freshwater. Interestingly, in one study *An. atroparvus* larvae were also found in used tires [49]. This species was generally recorded in rural sites, away from populated areas [50, 51] but some authors reported its occurrence also near human settlements [36] (Table 2).

#### Resting/overwintering behavior

*Anopheles atroparvus* is reported to hibernate as adult females and is known to have a different overwintering behavior as compared to other species of *An. maculipennis* s.l. It has been described to occasionally feed on blood if the shelters were relatively warm, but without egg production [4]. No other recent observations were found regarding this behavior, but it was assumed that its occurrence might be related to the presence of suitable winter resting sites where winter feeding is still possible, such as farms and animal shelters/nests [35, 44].

#### Feeding behavior

*Anopheles atroparvus* has been considered to be zoophilic or more specifically mammalophilic [80], with anecdotal reports of feeding events on humans [52]. This statement is in contrast with the definition of this species as a major malaria vector in some areas. For this reason, recent studies have attempted to clarify this aspect. Brugman et al. [53–55] reported the collection of a small number of *An. atroparvus* females during human landing catches conducted in the UK. Human blood was also found in a single field-collected female [56], while most studies have reported collections of mosquitoes that fed on a wide range of farming or wild animals [56–58]. Noteworthy, three articles reported detecting mosquitoes that had several blood meals on birds (chickens, stove doves and 1 blackbird) [54, 59, 60]. Little evidence is available before 2000 that clarified if *An. atroparvus* is endo- or exo-phagic, and only a single recent study reported that this species fed on animals located outdoors using human-made shelters for indoor resting after feeding [61] (Table 3).

#### Vector competence

Few older studies investigated the vector competence of *An. atroparvus* for tropical *Plasmodium*, but in all of these studies the authors concluded that this species was unable to transmit tropical strains of both Asian and African *Plasmodiium falciparum* [62–65], while in the past it was competent in supporting European strains [66]. However, this species has been demonstrated to be competent for non-local *Plasmodium vivax* and *Plasmodium malariae* strains [64]. In addition, it has been speculated that *An. atroparvus* was involved as a vector in an autochthonous case of *Plasmodium ovale* that occurred in central Spain, although the possibility of an airport malaria case was also considered due to the proximity of the patient’s residence to two international airports [67] (Table 4). In conclusion, all of the recent findings confirm that, given the mostly zoophilic nature of *An. atroparvus*, its role as a potential malaria vector can be considered low (Table 5).

**Table 4:** Competence of potential malaria vector species to *Plasmodium* species and their involvement in local transmission events according to the available literature

Species	*Plasmodium* species (tropical strains)	Competence tested	Post-eradication autochtonous cases (Europe, Middle East)	References
*An. atroparvus*	*P. falciparum*	No		[62–65, 337]
	*P. vivax*	Yes		[64]
	*P. malariae*	Yes		[64]
	*P. ovale*	No	Suspected	[64, 67]
*An. hyrcanus* s.l.	*P. falciparum*	No		[83]
	*P. vivax*	Yes	Suspected	[19, 83]
	*P. malariae*	Unknown		
	*P. ovale*	Unknown		
*An. labranchiae*	*P. falciparum*	Low		[62, 63, 87, 90, 97]
	*P. vivax*	Yes	Yes	[95–97]
	*P. malariae*	Unknown		
	*P. ovale*	Unknown		
*An. maculipennis* s.s.	*P. falciparum*	Unknown		
	*P. vivax*	Suspected	Suspected	[112–114]
	*P. malariae*	Unknown		
	*P. ovale*	Unknown		
*An. messeae/daciae*	*P. falciparum*	No		[10, 39, 64, 121]
	*P. vivax*	Low	Suspected	[10, 64, 123]
	*P. malariae*	Unknown		
	*P. ovale*	Unknown		
*An. plumbeus*	*P. falciparum*	Yes	Suspected	[155, 158, 159]
	*P. vivax*	Yes	Yes	[157, 158]
	*P. malariae*	Unknown		
	*P. ovale*	Unknown		
*An. sacharovi*	*P. falciparum*	Suspected		[64]
	*P. vivax*	Yes	Yes	[64, 107, 134–137]
	*P. malariae*	Unknown		
	*P. ovale*	Unknown		
*An. superpictus*	*P. falciparum*	Unknown		
	*P. vivax*	Yes	Suspected	[10, 137, 140]
	*P. malariae*	Unknown		
	*P. ovale*	Unknown

**Table 5:** Relative importance of potential malaria vector species based on current information, in particular *Plasmodium* competence and anthropophily

Species	Known competence	Anthropophily	Importance as potential malaria vector
*An. atroparvus*	*P. vivax*, *P. malariae* (*P. ovale*)	Low	Low
*An. hyrcanus* s.l.	*P. vivax*	High	High
*An. labranchiae*	*P. vivax*, (*P. falciparum*)	Opportunistic	High
*An. maculipennis* s.s.	(*P. vivax*)	Low	Moderate
*An. messeae/daciae*	(*P. vivax*)	Low	Moderate
*An. plumbeus*	*P. falciparum*, *P. vivax*	High	High
*An. sacharovi*	*P. vivax*, (*P. falciparum*)	Opportunistic	High
*An. superpictus*	*P. vivax*	Low	Low

For* Plasmodium* species presented in parenthesis the competence is uncertain

### *Anopheles hyrcanus *s.l.

*Anopheles hyrcanus* is considered to be a mosquito group that includes about 30 species distributed throughout the Palearctic and Oriental regions. In the last few years, the classification of these species has been subjected to debate. *Anopheles hyrcanus* s.s. Pallas, 1771 and *An. pseudopictus* Grassi, 1899 were formerly considered to be two distinct species occurring in Europe [19], but results from more recent studies based on genetic analyses suggest that *An. hyrcanus* s.s. and *An. pseudopictus* actually belong to the same taxon [20]. Therefore, we refer to *An. hyrcanus* s.l. when describing the bionomic features of this mosquito.

#### Occurrence

In France, *An. hyrcanus* s.l. is still as abundant as in the past, and it is currently considered to be the main malaria vector species [30, 33, 34, 68–70]. In Turkey, this species has been well described only in the last few years [20, 71–73]. Several studies, particularly those conducted after 2000, had the specific aim of identifying *An. hyrcanus* s.l., notably studies in Croatia, Hungary, Austria, Czech Republic and Slovakia [74]. A single study also reported the presence of *An. hyrcanus* s.l. in the Dagestan Lowland of Russia [75] (Table 1; Fig. 4).

#### Breeding sites

Traditionally, the preferred breeding sites for larval development in *An. hyrcanus* s.l. have been reported to be sun-heated stagnant water bodies rich in aquatic vegetation and vertical structures, such as reeds (e.g. floodplains and marshes) [76]. The same sites were reported in the articles extracted in this systematic review, in particular swamps, medium-large ponds and rice fields (Table 2).

High densities of *An. hyrcanus* s.l. were reported in the Camargue (France), where rice fields are predominant and considered to be the main breeding sites for this species [33, 34, 68, 70, 77, 78]. In particular, Tran et al. [77] reported that large populations of *An. hyrcanus* s.l. were frequently associated with rice-growing areas. However, in the same study, the authors also indicated that “larvae were also reported in other biotopes, such as reed beds and marshes with *Scirpus* […] this result is of great importance in explaining the presence of this species in the area even when rice paddies are dry (end of summer and autumn)”. In agreement with this evidence, *An. hyrcanus* s.l. larvae reported in countries of central and southeastern Europe were found mainly in large water collections, such as pools, fishponds and swamps [37, 79–82].

#### Resting/overwintering behavior

*Anopheles hyrcanus* s.l. adults are exophilic, with outdoor resting sites during the day. Gadzhieva [75] made detailed observations on the resting behavior of this mosquito, noting that females remained in indoor sites (houses and cowsheds) only for the day following the blood meal, while males and females at different physiological stages rested longer outdoors among the vegetation. In particular, the resting behavior of *An. hyrcanus* s.l. was influenced by environmental humidity; consequently, it is a species for which the proximity of the breeding sites is linked to the hygrophilous vegetation.

Diapausing females were observed to mainly exploit natural shelters, including clefts in the ground, grasses and reed bushes, while indoor-resting specimens were rarely observed [83].

#### Feeding behavior

*Anopheles hyrcanus* s.l. has historically been described as strictly mammalophilic, with a high degree of anthropophily. This behavior is confirmed by several recent studies that performed human landing catches and used animal baited traps [33, 68, 70, 71]. In particular, Ponçon et al. [33] reported that “*Anopheles hyrcanus* presented a huge anthropophily with spectacular aggressiveness on humans: during this study, scientists underwent massive attacks from females of this species”. Moreover, Aldemir et al. [71] reported that this mosquito exhibited similar anthropophilic biting patterns both indoors and outdoors. Recent information also indicates that this species can be collected by traps baited with birds, although the numbers are very low compared to the number of mosquitoes caught in other traps baited with other animals, such as horses [68, 70] (Table 3).

#### Vector competence

In terms of its role as a vector, some species of the *An. hyrcanus* group are considered vectors of malaria parasites. In a number of studies, the authors assumed *An. hyrcanus* s.l. to be involved in malaria transmission, specifically in northern Afghanistan and in the Camargue region of France [19, 33, 84, 85]. In the countries of Central Asia, *An. hyrcanus* s.l. was considered to be a secondary malaria vector. Regarding vector competence, *An. hyrcanus* s.l. can easily be infected with *P. vivax*, while there is no evidence that it can transmit *P. falciparum* [83]. The only recent, but questionable information for Europe is from a study carried out in Austria, in which a pool of *An. hyrcanus* s.l. was found to be molecularly positive for *Plasmodium* sp. [86]. In conclusion, the potential role of this species in malaria transmission can be considered to be high (Tables 4, 5). As such, this species should be the focus of study in the contexts of high mosquito densities (e.g. at the end of summer) when, in association with its high anthropophily, it may potentially be involved in local transmission events.

### *Anopheles labranchiae* Falleroni, 1926

#### Occurrence

The extracted articles identified the typical distribution of *An. labranchiae* in Europe to be the Mediterranean and the warmest areas of Europe [4, 87] (Table 1; Fig. 5). Its present distribution is similar to that reported in the past, with this species still common in central Italy [31–35]. In addition, two studies [88, 89] reported its presence also in Sardinia, but with a more limited occurrence than in the past. In Corsica, a single survey conducted in 2008 [87] indicated that *An. labranchiae* maintains its former abundance. In addition to these findings, Boccolini et al. reported the presence of *An. labranchiae* in southern Italy and in new, hilly areas northeastward, indicating a potential expansion of the species area to new ecological settings [90, 91]. *Anopheles labranchiae* was found for the first time in Romania [92] as larvae in a natural pond. The molecular analysis of collected specimens showed a similarity of 96% to specimens from Italy, suggesting that this species was imported from that country, taking into consideration that many inhabitants of the area worked in Italy and came back to Romania during the summer.

#### Breeding sites

The biology of *An. labranchiae* has been described in several old and often not easily accessible scientific articles, with the typical larval habitats reported to be stagnant and slow-moving freshwater sites, in particular rice fields. Larvae were described as able to develop in every type of larval habitat, with a preference for those exposed to sunlight, but they were considered unable to tolerate high levels of organic or mineral pollution. In recent years, little research has been carried out on the characteristics of larval sites of this species. In the literature retrieved for this systematic review, most of the studies were carried out in central Italy, where the most described breeding sites were rice fields (Table 2), indicating that the occurrence of *An. labranchiae* in Italy is closely correlated to the presence of rice fields, with high abundance in the coastal areas, where higher temperatures contribute to their development [93, 94].

#### Resting/overwintering behavior

*Anopheles labranchiae* is described in the retrieved literature as hibernating at the adult stage in both complete and incomplete hibernation states. Occasional winter blood-feeding is also described. Reproductive activity starts again when temperatures reach 16 °C [87]. No more information is available from the recent literature for this species.

#### Feeding behavior

Females of this species are considered as one of the most aggressive human biter *Anopheles*, with mainly exophagic behavior, although attempts to enter the dwellings to bite the hosts have been described [4]. However, the retrieved data indicate that the feeding behavior of *An. labranchiae* is predominantly opportunistic, with biting of both humans and animals. In studies from Italy and Corsica, mosquito females were collected during human landing catches around sunset, but they were also found resting in animal shelters. Analysis of the blood meals of sampled specimens revealed feedings on multiple animals, with the choice of host dependent on its availability [87, 93, 94] (Table 3).

#### Vector competence

In the past, *An. labranchiae* was considered to be the main malaria vector in the Mediterranean region, especially in malaria endemic areas during the first half of twentieth century, with studies showing naturally infected mosquitoes with high sporozoite indices. However, experimental assays of infection on *An. labranchiae* populations from central Italy and Corsica showed a very low susceptibility to tropical *P. falciparum* (laboratory strain NF54) [86, 89]. On the contrary, the competence of this species for natural strains of *P. vivax* seems possible. In fact, *An. labranchiae* has been implicated in autochthonous cases of malaria by *P. vivax* that have occurred both in Corsica and Italy [95–97], indicating that this species can still be considered a potential threat of malaria transmission in Europe (Tables 4, 5).

### *Anopheles maculipennis* s.s. Meigen, 1818

#### Occurrence

*Anopheles maculipennis* s.s. is probably the most widespread *Anopheles* species in Europe (Table 1; Fig. 6), even following the definition of *An. beklemishevi* Stegnii and Kabanova, 1976 as a new sibling species that replaced *An. maculipennis* s.s. in the northernmost areas of Europe [98]*.* In the most recent papers, *An. maculipennis* s.s. is confirmed as occurring all over Europe, with additional findings in north Italy [29, 32, 99] and Finland [100–102]. In particular, this species has been recorded in southeastern Europe [88, 103–106].

#### Breeding sites

*Anopheles maculipennis* s.s. can be found in different environments, ranging from coastal regions to inland areas. Larvae have been found both in clean waters and those moderately charged with organic matter, but there is as yet no evidence of its ability to exploit brackish water. The usual *An. maculipennis* s.s. breeding sites present vegetation or algae [104]. This species has a higher tolerance for moving water than other species of the *An. maculipennis* complex, with some data from recent studies reported in the retrieved literature confirming this tolerance (Table 2): larvae were frequently collected from streams, irrigation channels, river margins and, occasionally, in artificial lakes [50, 81, 107, 108]. As reported in the past, *An. maculipennis* s.s. was found at high altitudes and in hilly forested areas [109], reaching an altitude over 1500 m a.s.l. (Anatolia, Turkey [106]).

#### Resting-overwintering behavior

*Anopheles maculipennis* s.s. is known to be endophilic, resting in stables and dwellings. In recent literature, this species was reported to have been collected in large numbers in animal shelters, in particular those with pigs or sheep present [93, 110]. Diapause was described in older studies on this species, and no recent data are available. However, feeding activity and oviposition are described to be still possible during the winter, but stopped at lower temperatures [10].

#### Feeding behavior

*Anopheles maculipennis* s.s. is considered to be strongly zoophilic and, therefore, to play a secondary role in malaria transmission. However, in specific contexts (i.e. at high densities or where alternative hosts to humans are limited), the species has been found to show a certain degree of anthropophily [110]. Recent studies confirmed that animal blood meals are much more frequent, although some mosquitoes with human blood meals have been also found [93, 107, 111] (Table 3). Moreover, the authors of a paper reporting human landing catches carried out in Italy stated that “*An. maculipennis* s.s., although considered mainly zoophilic, also results as being very aggressive on humans during the night catches, both in presence or absence of animals” [93].

#### Vector competence

Among potential malaria vectors found in Europe, *An. maculipennis* s.s. is considered to be the mosquito with the lowest vectorial role in malaria transmission. Although no updated studies clarifying the real vector competence of this species for *P. vivax* or *P. falciparum*, in light of the recent information on its feeding behavior, *An. maculipennis* s.s. might be considered a potential malaria vector in some eastern and south-eastern European countries [112, 113] (Table 4). The occurrence of malaria in some areas where this species was abundant has been considered to be an evidence of its involvement in the maintenance of malaria transmission. The ability of *An. maculipennis* s.s to colonize different environments from sea level up to high altitudes, combined with its tolerance for cold temperatures, might balance its uncertain ability to support *Plasmodium* infection in some contexts [114]. Therefore, we suggest that this species be considered to be moderately worthy of attention in terms of potential involvement of malaria transmission events, at least in anthropized areas and areas with high mosquito densities (Table 5).

### *Anopheles messeae/daciae*

The species status of *An. messeae* has been debated for several years. Some authors suggest that it be split into two separated taxonomic units: *An. messeae* Falleroni, 1926 and *An. daciae* Linton, Nicolescu & Harbach, 2004 [17]. However, the hierarchical position of these taxa remains controversial. The existence of *An. daciae* as a distinct species was originally based on polymorphic sites at the ITS2 of the ribosomal DNA of *An. messeae* from Romania [17]. However, other authors consider this polymorphism to be an indicator of different forms/ecotypes that are consistent with the different geographical origins of the tested populations [32, 88, 115–117]. On the other hand, other authors have analyzed genomic differences and chromosomal inversions, especially on the* X*-chromosome, between *An. messeae* s.s. and *An. daciae* and argue that there is significant evidence for the diversification of the two lineages to be considered as distinct species [18, 118].

Following the definition of *An. daciae* as a new species, many studies (*n* = 26) assessed its occurrence in a number of countries, resulting in an increased number of studies focusing on *An. messeae* or *An. daciae*. However, the resulting literature was mainly based on the description of the molecular identification of *An. daciae*, with little additional information, and usually limited to reporting the location where the specimens were collected. Few articles reported ecological and biological differentiation between *An. messeae* and *An. daciae*, such as reproductive isolation, feeding behavior, vector competence, morphology, etc.

#### Occurrence

*Anopheles messeae/daciae* is one of the most widespread species of the *Anopheles maculipennis* complex in Europe (Table 1; Fig. 7). Its distribution ranges from Germany to Russia and to most parts of the Asian continent; it has not been reported in the Iberian Peninsula and France and is sporadically reported in southern Europe, with few records from Italy [29, 31, 32, 88, 99] and only one from Greece [103].

*Anopheles daciae* as a distinct species was identified first in Romania, then described using a molecular approach in Germany [41–44, 119–122], England and Wales [56, 123], the Czech Republic and Slovakia [39], Serbia [108] and more recently in Poland [124], Finland [101], Sweden [116] and Russia [18]. It has also been found in Italy and Belgium, with the provisional definition of *Anopheles daciae species inquirenda* [32, 117].

#### Breeding sites

Larvae of *An. messeae/daciae* have been found in many types of habitats, but most often in natural, forested areas with abundant vegetation, such as floating weeds or algae [89]. It seems to be a typical freshwater mosquito, preferring clean, oxygen-rich water with a relatively low content of dissolved ions [18, 81, 108, 113, 120]. There have been no reports of the presence of larvae in brackish sites, confirming the negative association with a certain degree of salinity (Table 2). Larvae have been found in ponds, swamps and small lakes [35, 43]; likewise, no collections in rice fields were recorded. Novikov and Vaulin [98] found *An. messeae/daciae* often associated with other species, attributing this observation to its wide ecological plasticity.

Few articles have reported distinct data for *An. messeae* or *An. daciae* in terms of breeding sites [18, 43, 108, 118, 121]. Both species were found mainly in the zone of temperate deciduous and mixed forests, with *An. messeae* negatively correlated with agricultural areas and *An. daciae* negatively correlated with pastures [43, 121]. Naumenko et al. [18] described *An. messeae* breeding sites to be associated with oxygen-rich (1.8–4.0 mg/l) waters, while *An. daciae* was found in atypical breeding sites, in waters with low oxygen content (0.8 mg/l).

#### Resting/overwintering behavior

Adults of *Anopheles messeae/daciae* have been found resting outdoors and to be particularly abundant in animal stocks, which is in agreement with the strong relationship between the high abundance of this species in rural/natural habitats and the presence of animal shelters in these areas [108, 113, 125]. Females were also collected in human dwellings, but in low numbers [125, 126], indicating that this species might also be present in anthropized contexts.

New information on *An. messeae/daciae* diapause was not retrieved. Based on previous descriptions, during the winter this species enters into full hibernation at the adult stage without any feeding activity, resting in enclosed shelters or buildings [4].

#### Feeding behavior

This species was described in the past as a strongly zoophilic feeder. The authors of one article stated that *An. messeae/daciae* does not feed on humans and, therefore, considered it to be a malaria vector of negligible importance [35]. Several recent studies have indicated that this species is an opportunistic feeder (Table 3). *Anopheles messeae/daciae* was captured in animal housing that sheltered different animal hosts, such as cows, horses, sheep and goats, but the results of this study did not unequivocally demonstrate whether this was a consequence of sampling bias or host choice [108]. Similarly, other studies repoorted that the blood meal analysis demonstrated feeding events on several host species, such as birds, deers, humans, horses and goats, with no particular preference [54, 56, 111, 125]. In other articles the identification of *An. messeae/daciae* blood meals indicated cattle and dog as major hosts [58, 127]. It was also recorded that females of this species are also attracted by humans [126], and this host-seeking behavior was also confirmed in a study using human landing catches [53].

Considering *An. messeae* and *An. daciae* to be separate species, limited and inconclusive evidence can be obtained in the retrieved literature on their respective feeding pattern. According to Danabalan et al. [56]*, An. daciae* may feed on animals and humans, whereas *An. messeae* s.s. appears to be strictly zoophilic. However, these observations were based on a single study that collected only a few samples and performed in a small area. Other authors speculated that *An. daciae* is more anthropophilic than *An. messeae* because it was found more frequently in larval habitats located close to human dwellings [118]. On the other hand, in a study carried out in two German zoological gardens to determine the feeding pattern of different mosquito species, *An. daciae* blood-fed only on wild mammals, with no human blood detected, while *An. messeae* blood-fed on both birds and mammals, humans included [111].

#### Vector competence

Available information indicates that the role of *An. messeae/daciae* as a malaria vector remains controversial. It seems refractory to tropical *P. falciparum* strains (even if a successful infection with a strain from the Central African Republic was obtained [64]), but it is considered to be a potential vector of *P. vivax* in northwestern Europe [10, 64]. In addition, based on old published articles, it is considered a potentially important vector in eastern Europe and western Asia [4]. Linton et al. [123] attempted to link the distribution of malaria cases in eastern Europe to the occurrence of the two tentative taxa of *An. messeae/daciae.* According to these authors, the different distribution of malaria cases might be an indication of the vector competence of both *An. messeae* and *An. daciae*, considering them as separate species with a different ecology and geographic distribution. The authors suggested that malaria cases that occurred in the past in Eurasia reflected the widespread distribution of *An. daciae* in this area and, consequently, they asserted that this taxon can be a better vector of *P. vivax* than *An. messeae* [39, 121]. Considering the lack of agreement on the existence of these two taxa, as well as the lack of recent information on vector competence for *Plasmodium* species of different populations of *An. messeae/daciae*, it is still difficult to assess its potential role in malaria transmission in Europe, but it should be cautiously considered as moderate (Tables 4, 5).

### *Anopheles sacharovi* Favre, 1903

#### Occurrence

Of all the *Anopheles* species recorded in Europe, *An. sacharovi* has the most southern distribution (Table 1; Fig. 8) and is considered to be the most threatening malaria vector across southeastern Europe and the Middle East. Despite anti-vector campaigns implemented across the eastern part of Europe, this species remains abundant. As in the past, this species is currently frequently detected in Greece and Turkey. Conversely, *An. sacharovi* seems to have disappeared from islands of Corsica and Sardinia, and only two doubtful findings have been reported in mainland Italy [29, 128].

#### Breeding sites

Larvae of *An. sacharovi* can develop in different water collections, usually those containing freshwater but also in brackish-salt water collections with a salinity of up to 20%, and it can tolerate a wide range of temperatures up to 38–40 °C [107, 129, 130]. *Anopheles sacharovi* is generally found in any kind of habitat containing horizontal vegetation, such as swamps, marshes, river margins, streams, pools and ditches. The retrieved literature confirmed the occurrence of larvae mainly in natural and large water collections, but also in irrigation channels, with all water collections characterized by abundant vegetation. However, compared to the past records, *An. sacharovi* currently seems to be less frequently detected in rice fields, probably due to the introduction of pesticide treatments (Table 2).

#### Resting/overwintering behavior

In terms of adult feeding behavior, *An. sacharovi* has been principally described as endophagic. It tends to rest in all types of dwellings, including both animal shelters and houses [131, 132]. Adult hibernation is incomplete, with periodic feeding events during the winter. This behavior is probably due to the distribution of this species in areas with rather mild winter temperatures. However, oviposition during hibernation was not mentioned in any study included in this systematic review.

#### Feeding behavior

Among the *Anopheles* species described in this review, *An. sacharovi* was considered to be the most anthropophilic, as confirmed in the recent literature, although several papers reported a certain degree of opportunistic feeding behavior. Generally, mosquitoes that were found in animal shelters had had a blood meal on the species stabled in the shelter [110, 112], but exophagic activity was also observed. Kampen et al. [107] reported that 23 out of 24 resting *An. sacharovi* females collected in sheds had blood-fed on an animal (mainly goat), with only one female having had a blood meal on a human. Similarly, Tavşanoğlu and Çağlar [133] stated that *An. sacharovi* fed preferably on animals rather than humans even if mosquitoes were collected in houses. However, the results of these studies were affected by the proximity of the houses and animal sheds, both usually being open during the summer, thereby allowing free movement of blood-fed females searching for a suitable resting place (Table 3).

#### Vector competence

The role of *An. sacharovi* as a primary malaria vector is partially due to its wide distribution and densities, in addition to its high ecological plasticity at both the adult and larval stages. In southeastern Europe and the Middle East, most malaria cases were reported to be caused by *P. vivax*, with one of the primary vectors being *An. sacharovi* [134]. In support of this finding, the authors of some studies reported autochthonous malaria cases that occurred in Greece in 1994–1995 and 2009–2010, although the country was declared to be free of malaria in 1974. Following these events, the entomological investigations indicated *An. sacharovi* as the predominant mosquito (up to 80%) in those areas [107, 135, 136], despite *Plasmodium* never being detected in field mosquitoes. However, this species was demonstrated to be experimentally competent not only for *P. vivax* [137] but also for tropical strains of *P. falciparum* [64], indicating its major importance as a malaria vector in Europe (Tables 4, 5).

### *Anopheles superpictus* Grassi, 1899

#### Occurrence

There are suggestions in the extracted literature that *An. superpictus* be considered a species complex [4]. Its wide distribution, ranging from the Mediterranean region to Southeast Asia, is probably the consequence of the distribution of different species partially overlapping in different geographic areas. However, no recent studies are available to confirm this hypothesis. Information on the biology of *An. superpictus* described in older publications refers to studies conducted outside of Europe [4]. All *An. superpictus* findings in the recent literature came from southeastern Europe, except for one study in Italy [138] where a few specimens were found on several occasions between 2011 and 2014 (Table 1; Fig. 9).

#### Breeding sites

Typically, the breeding sites of *An. superpictus* are temporary pools formed by fluctuations in the water level of rivers or streams caused by rain activity. Such sites are typically described as pools of clean water with gravelly riverbeds exposed to sunlight [4]. Several studies reported the presence of *An. superpictus* in man-made habitats, such as irrigation channels, rice fields, ditches and artificial pools [139]. Other larval samplings, such as those described by Yavaşoglu et al. [55, 140], were ponds, streams and swamps, while Violaris et al. [141] collected larvae from streams, slow-running waters, sunny seepages and irrigation systems. The ability of this species to tolerate a certain degree of salinity or pollutants is unclear. Among the literature examined in this review, only one publication reported larvae of *An. superpictus* in breeding sites with low salinity and a chloride concentration ranging between 30 and 96 mg/l [107] (Table 2).

#### Resting/overwintering behavior

No information is available in the literature on the overwintering behavior of *An. superpictus.* The only records retrieved report data on resting females, which were often caught in animal stables and houses in rural areas [72, 107, 140, 142].

#### Feeding behavior

Data on the feeding behavior of *An. superpictus* are also not exhaustive, and no other recent information was found. This mosquito species is mostly considered to be zoophilic and exophagic, despite questionable data were reported in literature. According to some authors, humans are a secondary host and attacked only in the absence of farm animals, while other authors suggest that *An. superpictus* can bite both humans and animals without preference, especially in periods of high densities, such as in late summer [4, 10] (Table 3).

#### Vector competence

The role of *An. superpictus* as a vector of *Plasmodium* is unclear. Older studies reported the susceptibility to *P. vivax* [137] and the association between high mosquito densities and malaria outbreaks [10]. Only one recent paper confirmed these findings, with the authors stating “*An. superpictus* occurred in Savur (Turkey) where there was an important malaria focus” [140]; however, the information might simply be a consequence of a spurious association (Table 4). At most, the available evidence indicates that this species might have a secondary role in the maintenance of malaria infection in areas where it reaches significant densities, such as in some areas of southeastern Europe (Table 5).

### *Anopheles plumbeus* Stephens, 1828

#### Occurrence

*Anopheles plumbeus* is one of the most diffused species among those targeted in this review. It is widespread throughout Europe up to the Caucasus and Asia, from colder to warmer climates (Table 1; Fig. 10).

#### Breeding sites

This species is known to be associated to a sylvatic environment, especially forested areas, breeding in water-filled tree holes, wood stocks and ground holes characterized by high concentrations of organic substances and oxygen deficiency. More recently, a shift of habitats has been observed for this species, from forested to anthropic ones, such as parks and cemeteries, where larvae have been found developing in holes in beeches and poplars [40, 50, 143]. In the last decade, another habitat adaptation was observed for this species: *An. plumbeus* was found to exploit artificial containers with stagnant water that had a composition similar to that of water in tree holes due to organic contamination. In fact, almost all of the recent papers retrieved for this review reported larval samplings of *An. plumbeus* in artificial containers. Larvae were found in tires, manure collection tanks and any other kind of man-made artificial container, including *Aedes* monitoring ovitraps [143–151]. In these artificial containers, mosquitoes not only found appropriate alternative breeding sites but also a lot of space for mass development [143, 152]. This shift in habitats has allowed the colonization of new areas and the increase in local densities (Table 2).

#### Resting/overwintering behavior

No data are currently available on the resting and diapausing activity of this mosquito. The only record found reported *An. plumbeus* larvae in England during all months of the year, while adults were found from late April to early October [126]. This finding leads to the hypothesis that this species is adapted to pass cold months as larvae, which will be a unique example among the species here described.

#### Feeding behavior

In the past, *An. plumbeus* was described to have a feeding activity at any time of the day, even in daylight, biting humans with persistence and aggressiveness both in urban and forested areas [153]. This behavior has been confirmed in recent studies, in which *An. plumbeus* was reported to bite during the daytime, in particular during the early and late hours of the day [53, 80]. In addition, it was particularly aggressive in attacking humans when mass development occurred, causing a serious nuisance [152, 154]. *Anopheles plumbeus* can also feed on other hosts, such as horses, wild animals and, more rarely, birds [68, 79, 111, 155] (Table 3).

#### Vector competence

Some laboratory studies reported that *An. plumbeus* is able to produce sporozoites of tropical strains of *P. falciparum* [156], as well as of *P. vivax* [157]. More recently, the vector competence of *An. plumbeus* for these two *Plasmodim* species was confirmed [158]. Also, a retrospective study speculated about its possible involvement in two cases of autochthonous *P. falciparum* malaria that occurred in Germany [159] (Table 4). In conclusion, despite *An. plumbeus* never being included as a potential malaria vector in past reviews, it is a species that may play a primary role in malaria transmission (Table 5), in particular in light of its anthropophily and the ability to exploit artificial containers as larval sites, allowing closer contact of this mosquito with humans.

## Conclusions

This systematic review provides an updated overview of the occurrence, ecology and vector competence of* Anopheles* mosquito species recorded in Europe that are historically associated with malaria transmission and/or currently potentially involved in autochthonous cases. This information will be useful for identifying the most important species in the current European epidemiological scenario.

The retrieved literature indicates a limited research interest in the targeted mosquito taxa, probably due to the very low number of malaria cases that occur in Europe. This lack of interest is supported by the main subject of the articles retrieved, with most dealing with the determination of the culicid fauna in a specific area or the vector surveillance of pathogens other than *Plasmodium*, such as viruses or filarial nematodes. However, a reasonable number of recent publications specifically addressed *Anopheles* and, in particular, attempted to clarify the presence of some species, such as *An. messeae/daciae* or *An. hyrcanus/pseudopictus*. In this regard, the development and implementation of molecular techniques for species identification have made a significant and noteworthy contribution, and is a factor that was not stressed in studies published before 2000.

**Fig. 11:**
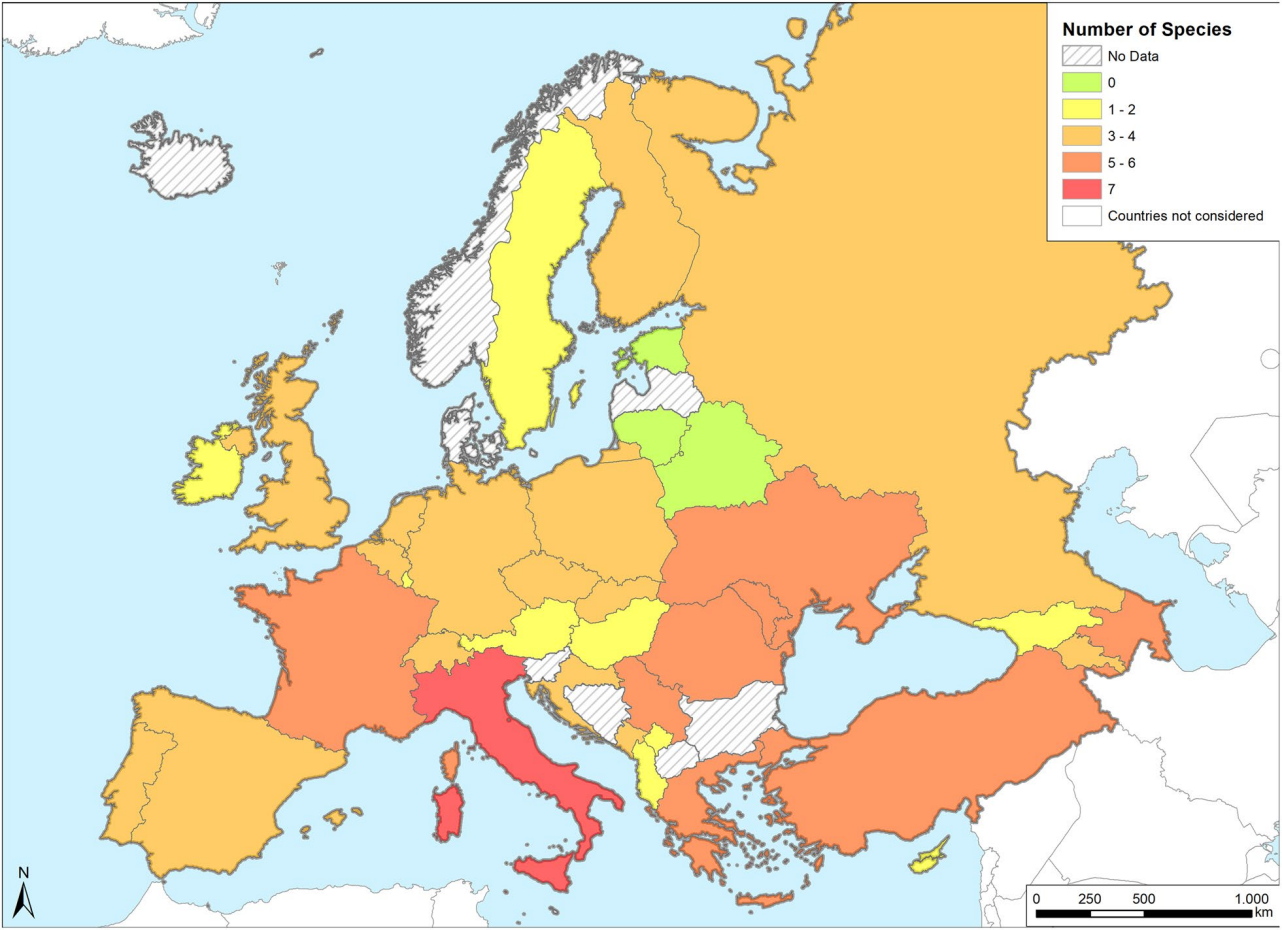
Number of reported potential malaria vector species per country. Green areas (Latvia and Belarus) show the absence of vectors due to the lack of information on species identification or reported information on a specific species only, such as the *An. maculipennis* complex

All of the *Anopheles* species targeted in this systematic review are considered to be potential vectors of malaria (*An. atroparvus*, *An. sacharovi*, *An. labranchiae*, *An. messeae/daciae*, *An. maculipennis* s.s., *An. superpictus*, *An. hyrcanus* s.l. and *An. plumbeus*) and are still present in European countries. In some areas of Europe they are also abundant in terms of species diversity, particularly in southern Europe (Fig. 11).

A north-to-south gradient of the presence of the targeted *Anopheles* mosquitoes is quite evident, indicating that, as expected, the majority of potential malaria vectors is mostly linked to the warm Mediterranean climate. Among all of the countries investigated, Italy is the only one in which more than six potential malaria vectors are found. This is not particularly surprising, considering the great diversity in habitats available in Italy, which is already exploited by one of the largest Culicidae fauna of the European continent (currently 64 species described [160], with several invasive species now established in the country [161]).

Despite this abundance, at the ecological level a reduction in their original habitats has been observed, as compared to the past (in particular for *An. sacharovi*). This reduction is mostly due to human interventions that modified the original larval habitats (wetlands in particular, which are currently mainly preserved nature areas of interest), and to the increasing usage of insecticides. Several *Anopheles* species (e.g. *An. atroparvus*, *An. labranchiae* and *An. maculipennis* s.s.) now exploit alternative larval habitats linked to some agricultural practices (e.g. rice cultivation and the use of extensive irrigation networks) that have increased in recent years, thereby allowing for the presence of mosquito populations also in anthropized areas. In particular, anthropic habitats are now exploited by new potential malaria vectors, as shown by the presence of *An. plumbeus*, which is now able to exploit urban environments using small water containers rich in organic matter as habitats. This indicates that some potential malaria vectors possess a non-negligible ecological plasticity.

Recent literature shows that most records of field collections involving potential malaria vectors are derived from arbovirus surveillance programs in which the CDC light trap was used as a standard method. Targeted *Anopheles* species, when specifically sampled, are mostly collected manually as resting adults, indicating a substantial lack of standardization in the monitoring of the *Anopheles* target species. In fact, among the trapping methods available in literature, only a few studies involving European potential malaria vectors compared the traps used [59, 78, 162, 163]. This lack of knowledge stresses the need to develop appropriate sampling strategies for these species in order to evaluate their distribution and abundance and, therefore, the appropriate surveillance programs in case of need.

A major limitation in the evaluation of epidemiological risks of local malaria transmission in Europe is the paucity of information on the competence of potential malaria vectors for tropical *Plasmodium* species. In fact, those studies demonstrating the capacity of the *Anopheles* species targeted in this review to transmit the different parasites can be considered to be outdated and fragmentary (Table 4).

One of the reasons for this lack of information is the difficulty to develop species-specific insectary breeding protocols, as those adopted for tropical malaria vectors are unfortunately inadequate. This is a limiting factor in obtaining the appropriate numbers of adult mosquitoes needed for competence testing, thus imposing the requirement that field-collected larvae be used as biological material, which in turn necessitates sorting and proper identification before testing. The only potential malaria vectors for which recent standardized procedures are available are *An. atroparvus* [164, 165], *An. labranchiae* [166], *An. superpictus* [166, 167] and *An. scaharovi* [166, 168], indicating that more efforts are needed to optimize rearing procedures for all potential malaria vectors.

Moreover, no information is available on the vector competence of any of the *Anopheles* species targeted in this review for *P. knowlesi*. Despite the rarity of imported human cases in Europe for this zoonotic *Plasmodium* and the uncertainty surrounding its capacity to produce significant gametocyte concentrations in humans [169], the increasing epidemiological evidence for the circulation of *P. knowlesi* in humans suggests that the possibility of potential malaria vectors locally transmitting this parasite needs to be taken into account.

Based on this review, the list of potential malaria vector species should be redefined in their relative importance (Table 5). Compared to previous literature [4], recent scientific evidence supports the addition of three new potential vectors in Europe: *An. maculipennis* s.s., *An. hyrcanus* s.l. and *An. plumbeus*. Even if the information on vectorial capacity of each species is subjected to local geographic/ecological differences, based on the available information we could consider *An. hyrcanus* s.l., *An. labranchiae*, *An. plumbeus* and *An. sacharovi* to be vectors of major importance, and *An. messeae/daciae* and *An. maculipennis* s.s. to be vector species of moderate importance, despite their large diffusion. In contrast, *An. atroparvus* and *An. superpictus* should be considered vectors of lower importance, especially given their low anthropophily. This revised classification may help define the current risk of re-introduction and emergence of autochthonous cases of malaria in different European countries, but also highlights the gaps still present in the knowledge of these vectorial systems, not only in terms of vector competence but also in the definition of appropriate sampling approaches. Further research on these potential malaria vectors is needed given recent climate, environmental and socio-economic changes that compel the adaptation of surveillance systems with the appropriate tools and knowledge for each species.

## Supplementary Information

**Additional file 1.** List of articles matching the eligibility criteria and assessed in this review (bibliographical details, list of potential vector species occurrence, monitoring/field activity, year of monitoring activity, identification method(s), sampling technique(s) and geographical details of sampling sites. When the presence of *Anopheles* species was not assigned to a specific site, all the sites monitored during that study were considered to be negative and marked as NA. *Abbreviation*: DD, Decimal degree; *NA*, not available.

## Data Availability

All data generated and analyzed in this review are included in this article, along with its additional files.

## References

[1] World Health Organization. Countries and territories certified malaria-free. https://www.who.int/malaria/areas/elimination/malaria-free-countries/en/. Accessed 30 Nov 2020.

[2] European Centre for Disease Prevention and Control (ECDC). Malaria—annual epidemiological report for 2019. Stockholm: ECDC; 2021. https://www.ecdc.europa.eu/en/publications-data/malaria-annual-epidemiological-report-2019. Accessed 30 Nov 2020.

[3] Sinka ME, Rubio-Palis Y, Manguin S, Patil AP, Temperley WH, Gething PW (2010). The dominant *Anopheles* vectors of human malaria in the Americas: occurrence data, distribution maps and bionomic précis. Parasit Vectors.

[4] Sinka ME, Bangs MJ, Manguin S, Coetzee M, Mbogo CM, Hemingway J (2010). The dominant *Anopheles* vectors of human malaria in Africa, Europe and the Middle East: occurrence data, distribution maps and bionomic précis. Parasites Vectors.

[5] Sinka ME, Bangs M, Manguin S, Theeraphap C, Patil AP, Temperley WH (2011). The dominant *Anopheles* vectors of human malaria in the Asia-Pacific region: occurrence data, distribution maps and bionomic précis. Parasit Vectors.

[6] White GB (1989). Malaria Geographical distribution of arthropod-borne diseases and their principal vectors.

[7] Kiszewski A, Mellinger A, Spielman A, Malaney P, Sachs SE, Sachs J (2004). A global index representing the stability of malaria transmission. Am J Trop Med Hyg.

[8] Sinka ME, Bangs MJ, Manguin S, Rubio-Palis Y, Chareonviriyaphap T, Coetzee M (2012). A global map of dominant malaria vectors. Parasites Vectors.

[9] Sinka ME, Pironon S, Massey NC, Longbottom J, Hemingway J, Moyes CL (2020). A new malaria vector in Africa: predicting the expansion range of *Anopheles stephensi* and identifying the urban populations at risk. Proc Natl Acad Sci USA.

[10] Jetten TH, Takken W (1994). Anophelism without malaria in Europe: a review of the ecology and distribution of the genus *Anopheles* in Europe. Wageningen Agric Univ Pap.

[11] Ramsdale C, Snow K (2000). Distribution of the genus *Anopheles* in Europe. Eur Mosq Bull.

[12] Kuhn KG, Campbell-Lendrum DH, Davies CR (2002). A continental risk map for malaria mosquito (Diptera: Culicidae) vectors in Europe. J Med Entomol.

[13] The Malaria Atlas Project (MAP). https://malariaatlas.org/. Accessed 30 Nov 2020.

[14] Beebe NW (2018). DNA barcoding mosquitoes: advice for potential prospectors. Parasitology.

[15] Liberati A, Altman DG, Tetzlaff J, Mulrow C, Gøtzsche PC, Ioannidis JPA (2009). The PRISMA statement for reporting systematic reviews and meta-analyses of studies that evaluate health care interventions: explanation and elaboration. PLoS Med.

[16] Hay SI, Sinka ME, Okara RM, Kabaria CW, Mbithi PM, Tago CC (2010). Developing global maps of the dominant *anopheles* vectors of human malaria. PLoS Med.

[17] Nicolescu G, Linton Y-M, Vladimirescu A, Howard TM, Harbach RE (2004). Mosquitoes of the *Anopheles maculipennis* group (Diptera: Culicidae) in Romania, with the discovery and formal recognition of a new species based on molecular and morphological evidence. Bull Entomol Res.

[18] Naumenko AN, Karagodin DA, Yurchenko AA, Moskaev AV, Martin OI, Baricheva EM (2020). Chromosome and genome divergence between the cryptic Eurasian malaria vector-species *Anopheles messeae* and *Anopheles daciae*. Genes.

[19] Ramsdale C (2001). Internal taxonomy of the Hyrcanus Group of *Anopheles* (Diptera: Culicidae) and its bearing on the incrimination of vectors of malaria in the west of the Palaearctic Region. Eur Mosq Bull.

[20] Ponçon N, Toty C, Kengne P, Alten B, Fontenille D (2008). Molecular evidence for similarity between *Anopheles hyrcanus* (Diptera: Culicidae) and *Anopheles pseudopictus* (Diptera: Culicidae), sympatric potential vectors of malaria in France. J Med Entomol.

[21] PubMed. https://pubmed.ncbi.nlm.nih.gov. Accessed 30 Nov 2021.

[22] Web of Science. https://apps.webofknowledge.com. Accessed 30 Nov 2021.

[23] Scopus. https://www.scopus.com/search/form.uri?display=basic#basic. Accessed 3 Feb 2022.

[24] Eurostat. Nomenclature of territorial units for statistics classification. https://ec.europa.eu/eurostat/web/nuts/background. Accessed 30 Nov 2020.

[25] Almeida APG, Galão RP, Sousa CA, Novo MT, Parreira R, Pinto J (2008). Potential mosquito vectors of arboviruses in Portugal: species, distribution, abundance and West Nile infection. Trans R Soc Trop Med Hyg.

[26] Capinha C, Gomes E, Reis E, Rocha J, Sousa CA, Do Rosário VE (2009). Present habitat suitability for *Anopheles atroparvus* (Diptera, Culicidae) and its coincidence with former malaria areas in mainland Portugal. Geospat Health.

[27] Lourenço PM, Sousa CA, Seixas J, Lopes P, Novo MT, Almeida APG (2011). *Anopheles atroparvus* density modeling using MODIS NDVI in a former malarious area in Portugal. J Vector Ecol.

[28] Romi R, Boccolini D, Di Luca M, La Rosa G, Marinucci M. Identification of the sibling species of the Anopheles maculipennis complex by heteroduplex analysis. Insect Mol Biol. 2000;9:509-13. 10.1046/j.1365-2583.2000.00213.x. 10.1046/j.1365-2583.2000.00213.x 11029669

[29] Talbalaghi A, Shaikevich E (2011). Molecular approach for identification of mosquito species (Diptera: Culicidae) in Province of Alessandria, Piedmont, Italy. Eur J Entomol.

[30] Vicente JL, Sousa CA, Alten B, Caglar SS, Falcutá E, Latorre JM (2011). Genetic and phenotypic variation of the malaria vector *Anopheles atroparvus* in southern Europe. Malar J.

[31] Zamburlini R, Cargnus E, Zandigiacomo P (2019). Mosquitoes (Diptera Culicidae) of Friuli Venezia Giulia (North-Eastern Italy): annotated checklist, geographical distribution and habitats of pre-imaginal stages. Redia.

[32] Calzolari M, Desiato R, Albieri A, Bellavia V, Bertola M, Bonilauri P (2021). Mosquitoes of the Maculipennis complex in Northern Italy. Sci Rep.

[33] Ponçon N, Toty C, L’Ambert G, Le Goff G, Brengues C, Schaffner F (2007). Biology and dynamics of potential malaria vectors in southern France. Malar J.

[34] Ponçon N, Toty C, L’Ambert G, Le Goff G, Brengues C, Schaffner F (2007). Population dynamics of pest mosquitoes and potential malaria and West Nile virus vectors in relation to climatic factors and human activities in the Camargue, France. Med Vet Entomol.

[35] Takken W, Geene R, Adam W, Jetten TH, van der Velden JA (2002). Distribution and dynamics of larval populations of *Anopheles messeae* and *A. atroparvus* in the delta of the rivers Rhine and Meuse, The Netherlands. Ambio.

[36] Bueno-Marí R, Bañeres AB, Peydró RJ (2014). Biodiversity and larval habitat heterogeneity of mosquitoes (Diptera: Culicidae) in Northern Spain. Acta Zool Bulg.

[37] Šebesta O, Gelbič I, Minář J (2012). Mosquitoes (Diptera: Culicidae) of the Lower Dyje River Basin (Podyjí) at the Czech-Austrian border. Open Life Sci.

[38] Šebesta O, Gelbič I, Peško J (2013). Seasonal dynamics of mosquito occurrence in the Lower Dyje River Basin at the Czech-Slovak-Austrian border. Ital J Zool.

[39] Blažejová H, Šebesta O, Rettich F, Mendel J, Čabanová V, Miterpáková M (2018). Cryptic species *Anopheles daciae* (Diptera: Culicidae) found in the Czech Republic and Slovakia. Parasitol Res.

[40] Čabanová V, Miterpáková M, Valentová D, Blažejová H, Rudolf I, Stloukal E (2018). Urbanization impact on mosquito community and the transmission potential of filarial infection in central Europe. Parasit Vectors.

[41] Kronefeld M, Dittmann M, Zielke D, Werner D, Kampen H (2012). Molecular confirmation of the occurrence in Germany of *Anopheles daciae* (Diptera, Culicidae). Parasit Vectors.

[42] Kronefeld M, Werner D, Kampen H (2014). PCR identification and distribution of *Anopheles daciae* (Diptera, Culicidae) in Germany. Parasitol Res.

[43] Kampen H, Schäfer M, Zielke DE, Walther D (2016). The Anopheles maculipennis complex (Diptera: Culicidae) in Germany: an update following recent monitoring activities. Parasitol Res.

[44] Lühken R, Czajka C, Steinke S, Jost H, Schmidt-Chanasit J, Pfitzner W (2016). Distribution of individual members of the mosquito *Anopheles maculipennis* complex in Germany identified by newly developed real-time PCR assays. Med Vet Entomol.

[45] Scholte EJ, Mars MH, Braks M, Den Hartog W, Ibanez-Justicia A, Koopmans M (2014). No evidence for the persistence of Schmallenberg virus in overwintering mosquitoes. Med Vet Entomol.

[46] Almeida APG, Freitas FB, Novo MT, Sousa CA, Rodrigues JC, Alves R (2010). Mosquito surveys and West Nile virus screening in two different areas of Southern Portugal, 2004–2007. Vector-borne Zoonotic Dis.

[47] Roiz D, Eritja R, Escosa R, Lucientes J, Marquès E, Melero-Alcíbar R (2007). A survey of mosquitoes breeding in used tires in Spain for the detection of imported potential vector species. J Vector Ecol.

[48] Sainz-Elipe S, Latorre J, Escosa R, Masià M, Fuentes M, Mas-Coma S (2010). Malaria resurgence risk in southern Europe: climate assessment in an historically endemic area of rice fields at the Mediterranean shore of Spain. Malar J.

[49] Bueno-Marí R, Jiménez-Peydró R (2012). Study of the malariogenic potential of Eastern Spain. Trop Biomed.

[50] Bueno-Marí R, Jiménez-Peydró R (2013). Anophelism in a former malaria area of northeastern Spain. J Arthropod Borne Dis.

[51] Roiz D, Ruiz S, Soriguer R, Figuerola J (2015). Landscape effects on the presence, abundance and diversity of mosquitoes in Mediterranean wetlands. PLoS ONE.

[52] Romi R, Pontuale G, Ciufolini MG, Fiorentini G, Marchi A, Nicoletti L (2004). Potential vectors of West Nile virus following an equine disease outbreak in Italy. Med Vet Entomol.

[53] Danabalan R, Monaghan MT, Ponsonby DJ, Linton Y-M (2014). Occurrence and host preferences of *Anopheles maculipennis* group mosquitoes in England and Wales. Med Vet Entomol.

[54] Brugman VA, England ME, Stoner J, Tugwell L, Harrup LE, Wilson AJ (2017). How often do mosquitoes bite humans in southern England? A standardised summer trial at four sites reveals spatial, temporal and site-related variation in biting rates. Parasit Vectors.

[55] Brugman VA, Hernández-Triana LM, England ME, Medlock JM, Mertens PPC, Logan JG (2017). Blood-feeding patterns of native mosquitoes and insights into their potential role as pathogen vectors in the Thames estuary region of the United Kingdom. Parasit Vectors.

[56] Brugman VA, Medlock JM, Logan JG, Wilson AJ, Lindsay SW, Fooks AR (2018). Bird-biting mosquitoes on farms in southern England. Vet Rec.

[57] Ventim R, Ramos JA, Osório H, Lopes RJ, Pérez-Tris J, Mendes L (2012). Avian malaria infections in western European mosquitoes. Parasitol Res.

[58] Hernandes-Traina LM, Brugman VA, Prosser SWJ, Weland C, Nikolova N, Thorne L (2017). Molecular approaches for blood meal analysis and species identification of mosquitoes (Insecta: Diptera: Culicidae) in rural locations in southern England, United Kingdom. Zootaxa.

[59] Roiz D, Roussel M, Munoz J, Ruiz S, Soriguer R, Figuerola J (2012). Efficacy of mosquito traps for collecting potential West Nile mosquito vectors in a natural Mediterranean wetland. Am J Trop Med Hyg.

[60] Martínez-de la Puente J, Muñoz J, Capelli G, Montarsi F, Soriguer R, Arnoldi D (2015). Avian malaria parasites in the last supper: identifying encounters between parasites and the invasive Asian mosquito tiger and native mosquito species in Italy. Malar J.

[61] Martínez-de la Puente J, Ruiz S, Soriguer R, Figuerola J (2013). Effect of blood meal digestion and DNA extraction protocol on the success of blood meal source determination in the malaria vector *Anopheles atroparvus*. Malar J.

[62] De Zulueta J, Ramsdale CD, Coluzzi M (1975). Receptivity to malaria in Europe. Bull World Health Organ.

[63] Ramsdale CD, Coluzzi M (1975). Studies on the infectivity of tropical African strains of *Plasmodium falciparum* to some southern European vectors of malaria. Parassitologia.

[64] Daskova NG, Rasnicyn SP (1982). Review of data on susceptibility of mosquitoes in the USSR to imported strains of malaria parasites. Bull World Health Organ.

[65] Ribeiro H, Batista JL, Ramos HC, Pires CA, Champalimaud JL, Costa JM (1989). An attempt to infect *An. atroparvus* from Portugal with African *Plasmodium falciparum*. Rev Port Doenç Infec.

[66] Van Dorp L, Gelabert P, Rieux A, De Manuel M, De-Dios T, Gopalakrishnan S (2020). *Plasmodium vivax* malaria viewed through the lens of an eradicated European strain. Mol Biol Evol.

[67] Cuadros J, Calvente MJ, Benito A, Arévalo J, Calero MA, Segura J (2002). *Plasmodium ovale* malaria acquired in Central Spain. Emerg Infect Dis.

[68] Balenghien T, Fouque F, Sabatier P, Bicout DJ (2006). Horse-, bird-, and human-seeking behavior and seasonal abundance of mosquitoes in a West Nile virus focus of southern France. J Med Entomol.

[69] Cailly P, Balenghien T, Ezanno P, Fontenille D, Toty C, Tran A (2011). Role of the repartition of wetland breeding sites on the spatial distribution of *Anopheles* and *Culex*, human disease vectors in southern France. Parasit Vectors.

[70] Poulin B, Lefebvre G, Muranyi-Kovacs C, Hilaire S (2017). Mosquito traps: an innovative, environmentally friendly technique to control mosquitoes. Int J Environ Res Public Health.

[71] Aldemir A, Bedir H, Demirci B, Alten B (2010). Biting activity of mosquito species (Diptera: Culicidae) in the Turkey-Armenia border area, Ararat Valley. Turkey J Med Entomol.

[72] Alkan SS, Aldemir A (2010). Seasonal dynamics of mosquitoes (Diptera: Culicidae) in animal barns and houses in Aras Valley, Turkey. Kafkas Univ Vet Fak Derg.

[73] Öncü C, Brinkmann A, Günay F, Kar S, Öter K, Sarıkaya Y (2018). West Nile virus, *Anopheles* flavivirus, a novel flavivirus as well as Merida-like rhabdovirus Turkey in field-collected mosquitoes from Thrace and Anatolia. Infect Genet Evol.

[74] Übleis SS, Cuk C, Nawratil M, Butter J, Schoener E, Obwaller AG (2018). Xenomonitoring of mosquitoes (Diptera: Culicidae) for the presence of filarioid helminths in Eastern Austria. Can J Infect Dis Med Microbiol.

[75] Gadzhieva SS (2007). Emergence and daytime resting sites in the malarial mosquito *Anopheles hyrcanus* (Culicidae) from the Daghestan lowland. Entomol Rev.

[76] Becker N, Petric D, Zgomba M, Boase C, Madon M, Dahl CH (2010). Mosquitoes and their control.

[77] Tran A, Ponçon N, Toty C, Linard C, Guis H, Ferré J-B (2008). Using remote sensing to map larval and adult populations of *Anopheles hyrcanus* (Diptera: Culicidae) a potential malaria vector in southern France. Int J Health Geogr.

[78] L’Ambert G, Ferré J-B, Schaffner F, Fontenille D (2012). Comparison of different trapping methods for surveillance of mosquito vectors of West Nile virus in Rhône Delta, France. J Vector Ecol.

[79] Šebesta O, Halouzka J, Hubálek Z, Juřicová Z, Rudolf I, Šikutová S (2010). Mosquito (Diptera: Culicidae) fauna in an area endemic for West Nile virus. J Vector Ecol.

[80] Šebesta O, Gelbič I, Peško J (2011). Daily and seasonal variation in the activity of potential vector mosquitoes. Open Life Sci.

[81] Sulesco TM, Toderas LG, Uspenskaia IG, Toderas IK (2015). Larval habitats diversity and distribution of the mosquito (Diptera: Culicidae) species in the Republic of Moldova. J Med Entomol.

[82] Zana B, Kemenesi G, Antal L, Foldes F, Oldal M, Banyai K (2017). Molecular traces of a putative novel insect flavivirus from *Anopheles hyrcanus* mosquito species in Hungary. Acta Virol.

[83] Zahar AR, World Health Organization, Division of Vector Biology and Control & World Health Organization, Malaria Action Programme. Vector bionomics in the epidemiology and control of malaria/prepared by A. R. Zahar. Pt. 1, The WHO African Region and the Southern WHO Eastern Mediterranean Region. Geneva: World Health Organization. 1985. https://apps.who.int/iris/handle/10665/62183. Accessed 30 Nov 2020.

[84] Onori E, Nushin MK, Cullen JE, Yakubi GH, Mohammed K, Christal F (1975). An epidemiological assessment of the residual effect of DDT on *Anopheles hyrcanus* s.l. and *An. pulcherrimus* (Theobald) in the North Eastern region of Afghanistan. Trans R Soc Trop Med Hyg.

[85] Faulde MK, Hoffmann R, Fazilat KM, Hoerauf A (2007). Malaria reemergence in northern Afghanistan. Emerg Infect Dis.

[86] Seidel B, Silbermayr K, Kolodziejek J, Indra A, Nowotny N, Allerberger F (2013). Detection of *Plasmodium* sp.-infested *Anopheles hyrcanus* (Pallas 1771) (Diptera: Culicidae) in Austria, 2012. Wien Klin Wochenschr.

[87] Toty C, Barré H, Le Goff G, Larget-Thiéry I, Rahola N, Couret D (2010). Malaria risk in Corsica, former hot spot of malaria in France. Malar J.

[88] Di Luca M, Boccolini D, Marinucci M, Romi R (2004). Intrapopulation polymorphism in *Anopheles messeae* (*An. maculipennis* complex) inferred by molecular analysis. J Med Entomol.

[89] Foxi C, Puggioni G, Meloni G, Rossi R, Rocchigiani AM, Vento L (2018). Entomological surveillance of Zika virus in Sardinia, Italy, 2016. Vet Ital.

[90] Boccolini D, Toma L, Luca MD, Severini F, Cocchi M, Bella A (2012). Impact of environmental changes and human-related factors on the potential malaria vector, *Anopheles labranchiae* (Diptera: Culicidae), in Maremma, Central Italy. J Med Entomol.

[91] Boccolini D, Menegon M, Di Luca M, Toma L, Severini F, Marucci G (2020). Non-imported malaria in Italy: paradigmatic approaches and public health implications following an unusual cluster of cases in 2017. BMC Public Health.

[92] Ivanescu M-L, Acatrinei D, Pavel I, Sulesco T, Miron L (2015). PCR identification of five species from the *Anopheles maculipennis* complex (Diptera: Culicidae) in North-Eastern Romania. Acta Parasitol.

[93] Di Luca M, Boccolini D, Severini F, Toma L, Barbieri FM, Massa A (2009). A 2-year entomological study of potential malaria vectors in Central Italy. Vector-Borne Zoonotic Dis.

[94] Romi R, Boccolini D, Vallorani R, Severini F, Toma L, Cocchi M (2012). Assessment of the risk of malaria re-introduction in the Maremma plain (Central Italy) using a multi-factorial approach. Malar J.

[95] Baldari M, Tamburro A, Sabatinelli G, Romi R, Severini C, Cuccagna G (1998). Malaria in Maremma, Italy. Lancet.

[96] Armengaud A, Legros F, Quatresous I, Barre H, Valayer P, Fanton Y (2006). A case of autochthonous *Plasmodium vivax* malaria, Corsica, August 2006. Euro Surveill.

[97] Romi R, Boccolini D, Menegon M, Rezza G (2012). Probable autochthonous introduced malaria cases in Italy in 2009–2011 and the risk of local vector-borne transmission. Euro Surveill.

[98] Novikov YM, Vaulin OV (2014). Expansion of *Anopheles maculipennis* s.s. (Diptera: Culicidae) to northeastern Europe and northwestern Asia: causes and consequences. Parasit Vectors.

[99] Tagliapietra V, Arnoldi D, Di Luca M, Toma L, Rizzoli A (2019). Investigation on potential malaria vectors (*Anopheles* spp.) in the Province of Trento, Italy. Malar J.

[100] Culverwell CL (2018). A report on the mosquitoes of mainland Åland, southwestern Finland and revised list of Finnish mosquitoes. Med Vet Entomol.

[101] Culverwell CL, Vapalahti OP, Harbach RE (2020). *Anopheles daciae*, a new country record for Finland. Med Vet Entomol.

[102] Culverwell CL, Uusitalo RJ, Korhonen EM, Vapalahti OP, Huhtamo E, Harbach RE (2021). The mosquitoes of Finland: updated distributions and bionomics. Med Vet Entomol.

[103] Linton Y-M, Samanidou-Voyadjoglou A, Harbach RE (2002). Ribosomal ITS2 sequence data for *Anopheles maculipennis* and *An. messeae* in northern Greece, with a critical assessment of previously published sequences. Insect Mol Biol.

[104] Linton Y-M, Smith L, Koliopoulos G, Samanidou-Voyadjoglou A, Zounos AK, Harbach RE (2003). Morphological and molecular characterization of *Anopheles* (*Anopheles*) *maculipennis* Meigen, type species of the genus and nominotypical member of the Maculipennis Complex. Syst Entomol.

[105] Simsek FM, Ulger C, Akiner MM, Tuncay SS, Kiremit F, Bardakci F (2011). Molecular identification and distribution of *Anopheles maculipennis* complex in the Mediterranean region of Turkey. Biochem Syst Ecol.

[106] Sevgili E, Simsek FM (2012). Distribution pattern and molecular identification of *Anopheles maculipennis* complex in eight river basins of Anatolia, Turkey. North West J Zool.

[107] Kampen H, Proft J, Etti S, Maltezos E, Pagonaki M, Maier WA (2003). Individual cases of autochthonous malaria in Evros Province, northern Greece: entomological aspects. Parasitol Res.

[108] Kavran M, Zgomba M, Weitzel T, Petric D, Manz C, Becker N (2018). Distribution of *Anopheles daciae* and other *Anopheles maculipennis* complex species in Serbia. Parasitol Res.

[109] Akiner MM, Ekşi E (2016). Impact of environmental factors on *Anopheles maculipennis* complex (Diptera: Culicidae) populations in three localities of Turkey. Int J Mosq Res.

[110] Linton Y-M, Smith L, Koliopoulos G, Zounos AK, Samanidou-Voyadjoglou A, Patsoula E (2007). The *Anopheles* (*Anopheles*) *maculipennis* complex (Diptera: Culicidae) in Greece. J Nat Hist.

[111] Heym EC, Kampen H, Schäfer M, Walther D (2019). Mosquito bloodmeal preferences in two zoological gardens in Germany. Med Vet Entomol.

[112] Romi R, Boccolini D, Hovanesyan I, Grigoryan G, Luca MD, Sabatinelli G (2002). *Anopheles sacharovi* (Diptera: Culicidae): a reemerging malaria vector in the Ararat Valley of Armenia. J Med Entomol.

[113] Dakic D, Kulisic Z, Stajkovic N, Pelemis M, Cobeljic M, Stanimirovic Z (2008). Ecology of *Anopheles* mosquitoes in Belgrade area: estimating vector potential for malaria retransmission. Acta Vet Beogr.

[114] Bruce-Chwatt LJ, de Zulueta J (1980). The rise and fall of malaria in Europe.

[115] Bezzhonova OV, Goryacheva II (2008). Intragenomic heterogeneity of rDNA internal transcribed spacer 2 in *Anopheles messeae* (Diptera: Culicidae). J Med Entomol.

[116] Lilja T, Eklöf D, Jaenson TGT, Lindström A, Terenius O (2020). Single nucleotide polymorphism analysis of the ITS2 region of two sympatric malaria mosquito species in Sweden: Anopheles daciae and *Anopheles messeae*. Med Vet Entomol.

[117] Smitz N, De Wolf K, Gheysen A, Deblauwe I, Vanslembrouck A, Meganck K (2021). DNA identification of species of the *Anopheles maculipennis* complex and first record of *An. daciae* in Belgium. Med Vet Entomol.

[118] Vaulin OV, Karagodin DA, Novgorodova TA, Glupov VV (2020). Analysis of *Anopheles messeae* s.l. intron gene polymorphism associated with imidacloprid resistance. J Vector Ecol.

[119] Weitzel T, Gauch C, Becker N (2012). Identification of *Anopheles daciae* in Germany through ITS2 sequencing. Parasitol Res.

[120] Kronefeld M, Kampen H, Sassnau R, Werner D (2014). Molecular detection of *Dirofilaria immitis*, *Dirofilaria repens* and *Setaria tundra* in mosquitoes from Germany. Parasit Vectors.

[121] Czajka C, Weitzel T, Kaiser A, Pfitzner WP, Becker N (2020). Species composition, geographical distribution and seasonal abundance of the *Anopheles maculipennis* complex along the Upper Rhine, Germany. Parasitol Res.

[122] Werner D, Kowalczyk S, Kampen H (2020). Nine years of mosquito monitoring in Germany, 2011–2019, with an updated inventory of German culicid species. Parasitol Res.

[123] Linton Y-M, Lee A, Curtis C (2005). Discovery of a third member of the Maculipennis group in SW England. J Eur Mosq Control Assoc.

[124] Rydzanicz K, Czułowska A, Manz C, Jawień P (2017). First record of *Anopheles daciae* (Linton, Nicolescu & Harbach, 2004) in Poland. J Vector Ecol.

[125] Fyodorova MV, Savage HM, Lopatina JV, Bulgakova TA, Ivanitsky AV, Platonova OV (2006). Evaluation of potential West Nile virus vectors in Volgograd region, Russia, 2003 (Diptera: Culicidae): species composition, bloodmeal host utilization, and virus infection rates of mosquitoes. J Med Entomol.

[126] Snow K, Medlock JM (2008). The mosquitoes of Epping Forest, Essex, UK. J Eur Mosq Control Assoc.

[127] Brugman VA, Hernández-Triana LM, Prosser SWJ, Weland C, Westcott DG, Fooks AR (2015). Molecular species identification, host preference and detection of myxoma virus in the *Anopheles maculipennis* complex (Diptera: Culicidae) in southern England, UK. Parasites Vectors.

[128] Möhlmann TWR, Wennergren U, Tälle M, Favia G, Damiani C, Bracchetti L (2017). Community analysis of the abundance and diversity of mosquito species (Diptera: Culicidae) in three European countries at different latitudes. Parasit Vectors.

[129] Artemiev MM. Anopheles mosquito—main malaria vectors in the USSR. In: International scientific project on ecologically safe methods for control of malaria and its vectors. International scientific project on ecologically safe methods for control of malaria and its vectors. 1980. Collected Lectures 2. p. 45–71.

[130] Sedaghat MM, Linton Y-M, Nicolescu G, Smith L, Koliopoulos G, Zounos AK (2003). Morphological and molecular characterization of *Anopheles* (*Anopheles*) *sacharovi* Favre, a primary vector of malaria in the Middle East. Syst Entomol.

[131] Yurttas H, Alten B (2006). Geographic differentiation of life table attributes among *Anopheles sacharovi* (Diptera: Culicidae) populations in Turkey. J Vector Ecol.

[132] Tavşanoğlu N, Çağlar SS (2008). The vectorial capacity of *Anopheles sacharovi* in the malaria endemic area of Şanlıurfa, Turkey. J Eur Mosq Control Assoc.

[133] Yavaşoglu Sİ, Fatih CÜ, Şimşek M (2021). The first implementation of allele-specific primers for detecting the knockdown and acetylcholinesterase target site mutations in malaria vector, *Anopheles sacharovi*. Pestic Biochem Physiol.

[134] Özbilgin A, Topluoglu S, Es S, Islek E, Mollahaliloglu S, Erkoc Y (2011). Malaria in Turkey: successful control and strategies for achieving elimination. Acta Trop.

[135] Danis K, Baka A, Lenglet A, Van Bortel W, Terzaki I, Tseroni M (2011). Autochthonous *Plasmodium vivax* malaria in Greece, 2011. Euro Surveill.

[136] Andriopoulos P, Economopoulou A, Spanakos G, Assimakopoulos G (2013). A local outbreak of autochthonous *Plasmodium vivax* malaria in Laconia, Greece—a re-emerging infection in the southern borders of Europe?. Int J Infect Dis.

[137] Kasap H (1990). Comparison of experimental infectivity and development of *Plasmodium vivax* in *Anopheles sacharovi* and *An. superpictus* in Turkey. Am J Trop Med Hyg.

[138] Mancini G, Montarsi F, Calzolari M, Capelli G, Dottori M, Ravagnan S (2017). Mosquito species involved in the circulation of West Nile and Usutu viruses in Italy. Vet Ital.

[139] World Health Organization (WHO). Mosquitoes of the genus Anopheles in countries of the WHO European Region having faced a recent resurgence of malaria. Regional research project, 2003–2007. 2008. WHO: Copenhagen. https://www.euro.who.int/__data/assets/pdf_file/0006/98763/E92010.pdf. Accessed 30 Nov 2020.

[140] Yavaşoglu Sİ, Yaylagül EÖ, Akıner MM, Ülger C, Çağlar SS, Şimşek FM (2019). Current insecticide resistance status in *Anopheles sacharovi* and *Anopheles superpictus* populations in former malaria endemic areas of Turkey. Acta Trop.

[141] Violaris M, Vasquez MI, Samanidou A, Wirth MC, Hadjivassilis A (2009). The mosquito fauna of the Republic of Cyprus: a revised list. J Am Mosq Control Assoc.

[142] Ergunay K, Gunay F, Erisoz Kasap O, Oter K, Gargari S, Karaoglu T (2014). Serological, molecular and entomological surveillance demonstrates widespread circulation of West Nile virus in Turkey. PLoS Negl Trop Dis.

[143] Dekoninck W, Hendrickx F, Van Bortel W, Versteirt V, Coosemans M, Damiens D (2011). Human-induced expanded distribution of *Anopheles plumbeus*, experimental vector of West Nile virus and a potential vector of human malaria in Belgium. J Med Entomol.

[144] Schaffner F, Van Bortel W, Coosemans M (2004). First record of *Aedes* (*Stegomyia*) *albopictus* in Belgium. J Am Mosq Control Assoc.

[145] Versteirt V, Schaffner F, Garros C, Dekoninck W, Coosemans M, Van Bortel W (2009). Introduction and establishment of the exotic mosquito species *Aedes japonicus japonicus* (Diptera: Culicidae) in Belgium. J Med Entomol.

[146] Romanović M, Merdić E (2011). Investigation of mosquito larvae (Diptera, Culicidae) in the coastal area of Dalmatia, Croatia. Period Biol.

[147] Townroe S, Callaghan A (2014). British container breeding mosquitoes: the impact of urbanisation and climate change on community composition and phenology. PLoS ONE.

[148] Balestrino F, Schaffner F, Forgia DL, Paslaru AI, Torgerson PR, Mathis A (2016). Field evaluation of baited traps for surveillance of *Aedes japonicus japonicus* in Switzerland. Med Vet Entomol.

[149] Martínez-Barciela Y, Martínez JMP, Torres MIS, Ortega ÁP, González JCO, González JG (2020). First records of *Anopheles* (*Anopheles*) *plumbeus* Stephens, 1828 and *Culex* (*Culex*) *torrentium* Martini, 1925 (Diptera: Culicidae) in Galicia (NW Spain). J Vector Ecol.

[150] Paronyan L, Babayan L, Manucharyan A, Manukyan D, Vardanyan H, Melik-Andrasyan G (2020). The mosquitoes of Armenia: review of knowledge and results of a field survey with first report of *Aedes albopictus*. Parasite.

[151] Heym EC, Kampen H, Fahle M, Hohenbrink TL, Schäfer M, Scheuch DE (2017). *Anopheles plumbeus* (Diptera: Culicidae) in Germany: updated geographic distribution and public health impact of a nuisance and vector mosquito. Trop Med Int Health.

[152] Früh L, Kampen H, Koban MB, Pernat N, Schaub GA, Werner D (2020). Oviposition of *Aedes japonicus japonicus* (Diptera: Culicidae) and associated native species in relation to season, temperature and land use in western Germany. Parasit Vectors.

[153] Shute PG (1954). Indigenous *P. vivax* malaria in London believed to have been transmitted by *Anopheles plumbeus*. Mon Bull Minist Health Public Health Lab Serv.

[154] Ibanez-Justicia A, Stroo A, Dik M, Beeuwkes J, Scholte EJ (2015). National mosquito (Diptera: Culicidae) survey in The Netherlands 2010–2013. J Med Entomol.

[155] Cerný O, Votýpka J, Svobodová M (2011). Spatial feeding preferences of ornithophilic mosquitoes, blackflies and biting midges. Med Vet Entomol.

[156] Eling W, van Gemert GJ, Akinpelu O, Curtis J, Curtis CF (2003). Production of *Plasmodium falciparum* sporozoites by *Anopheles plumbeus*. Eur Mosq Bull.

[157] Shute PG, Maryon M (1974). Malaria in England past, present and future. J R Soc Promot Health.

[158] Schaffner F, Thiéry I, Kaufmann C, Zettor A, Lengeler C, Mathis A (2012). *Anopheles plumbeus* (Diptera: Culicidae) in Europe: a mere nuisance mosquito or potential malaria vector?. Malar J.

[159] Krüger A, Rech A, Su X-Z, Tannich E (2001). Two cases of autochthonous *Plasmodium falciparum* malaria in Germany with evidence for local transmission by indigenous *Anopheles plumbeus*. Trop Med Int Health.

[160] Severini F, Toma L, Di Luca M, Romi R (2009). Le zanzare italiane: generalità e identificazione degli adulti (Diptera, Culicidae). Fragm Entomol.

[161] Montarsi F, Martini S, Michelutti A, Da Rold G, Mazzucato M, Qualizza D, et al. The invasive mosquito *Aedes japonicus japonicus* is spreading in northeastern Italy. Parasit Vectors. 2019;12(1):120. 10.1186/s13071-019-3387-x. 10.1186/s13071-019-3387-x PMC6434805 30909981

[162] Drago A, Marini F, Caputo B, Coluzzi M, Della Torre A, Pombi M (2012). Looking for the gold standard: assessment of the effectiveness of four traps for monitoring mosquitoes in Italy. J Vector Ecol.

[163] Lühken R, Pfitzner W, Börstler J, Garms R, Huber K, Schork N (2014). Field evaluation of four widely used mosquito traps in Central Europe. Parasit Vectors.

[164] Novikov YM (2016). Rearing of *Anopheles beklemishevi* (Diptera: Culicidae) and the possibility of its hybridization with *An. atroparvus* under laboratory conditions. J Vector Ecol.

[165] Birnberg L, Aranda C, Talavera S, Núñez AI, Escosa R, Busquets N (2020). Laboratory colonization and maintenance of *Anopheles atroparvus* from the Ebro Delta, Spain. Parasit Vectors.

[166] Coluzzi M (1964). Maintenance of laboratory colonies of *Anopheles* mosquitoes. Bull World Health Organ.

[167] Rasnitsyn SP, Yasyukevich VV, Zvantsov AB, Artemiev M (1990). Establishment of a laboratory colony of *Anopheles superpictus*. Med Parazitol.

[168] Rasnitsyn SP (1985). Massovoe kul'tivirovanie *Anopheles sacharovi* Favre [Mass cultivation of *Anopheles sacharovi* Favre]. Med Parazitol.

[169] Maeno Y, Culleton R, Quang NT, Kawai S, Marchand RP, Nakazawa S (2017). *Plasmodium knowlesi* and human malaria parasites in Khan Phu, Vietnam: gametocyte production in humans and frequent co-infection of mosquitoes. Parasitology.

[170] Török E, Kolcsár LP, Keresztes L (2019). New records and faunistic data of mosquitoes (Diptera, culicidae) from Albania, Hungary, Macedonia, Montenegro, and Serbia. Turk J Zool.

[171] Artemov GN, Velichevskaya AI, Bondarenko SM, Karagyan GH, Aghayan SA, Arakelyan MS (2018). A standard photomap of the ovarian nurse cell chromosomes for the dominant malaria vector in Europe and Middle East *Anopheles sacharovi*. Malar J.

[172] Lebl K, Nischler EM, Walter M, Brugger K, Rubel F (2013). First record of the disease vector *Anopheles hyrcanus* in Austria. J Am Mosq Control Assoc.

[173] Zittra C, Waringer J (2014). Species inventory, ecology, and seasonal distribution patterns of Culicidae (Insecta: Diptera) in the National Park Donau-Auen (Lower Austria). Aquat Insects.

[174] Silbermayr K, Eigner B, Joachim AL, Duscher GG, Seidel B, Allerberger F (2014). Autochthonous *Dirofilaria repens* in Austria. Parasit Vectors.

[175] Lebl K, Zittra C, Silbermayr K, Obwaller A, Berer D, Brugger K (2015). Mosquitoes (Diptera: Culicidae) and their relevance as disease vectors in the city of Vienna, Austria. Parasitol Res.

[176] Zittra C, Vitecek S, Obwaller AG, Rossiter H, Eigner B, Zechmeister T (2017). Landscape structure affects distribution of potential disease vectors (Diptera: Culicidae). Parasit Vectors.

[177] Namazov ND (2014). The distribution of mosquitoes (Diptera, Culicidae) in the Republic of Azerbaijan. Entomol Rev.

[178] Sulesco T, Volkova T, Yashkova S, Tomazatos A, von Thien H, Lühken R (2016). Detection of *Dirofilaria repens* and *Dirofilaria immitis* DNA in mosquitoes from Belarus. Parasitol Res.

[179] Versteirt V, de Clercq EM, Fonseca DM, Pecor J, Schaffner F, Coosemans M (2012). Bionomics of the established exotic mosquito species *Aedes koreicus* in Belgium, Europe. J Med Entomol.

[180] Boukraa S, Raharimalala FN, Zimmer J-Y, Schaffner F, Bawin T, Haubruge E (2013). Reintroduction of the invasive mosquito species *Aedes albopictus* in Belgium in July 2013. Parasite.

[181] Versteirt V, Boyer S, Damiens D, De Clercq EM, Dekoninck W, Ducheyne E (2013). Nationwide inventory of mosquito biodiversity (Diptera: Culicidae) in Belgium, Europe. Bull Entomol Res.

[182] Deblauwe I, Demeulemeester J, De Witte J, Hendy A, Sohier C, Madder M (2015). Increased detection of *Aedes albopictus* in Belgium: no overwintering yet, but an intervention strategy is still lacking. Parasitol Res.

[183] Versteirt V, Nagy ZT, Roelants P, Denis L, Breman FC, Damiens D (2015). Identification of Belgian mosquito species (Diptera: Culicidae) by DNA barcoding. Mol Ecol Resour.

[184] Boukraa S, de La Grandiere MA, Bawin T, Raharimalala FN, Zimmer J-Y, Haubruge E (2016). Diversity and ecology survey of mosquitoes potential vectors in Belgian equestrian farms: a threat prevention of mosquito-borne equine arboviruses. Prev Vet Med.

[185] Raharimalala FN, Boukraa S, Bawin T, Boyer S, Francis F (2016). Molecular detection of six (endo-) symbiotic bacteria in Belgian mosquitoes: first step towards the selection of appropriate paratransgenesis candidates. Parasitol Res.

[186] Wang L, Rosales Rosas AL, De Coninck L, Shi C, Bouckaert J, Matthijnssens J (2021). Establishment of *Culex modestus* in Belgium and a glance into the virome of Belgian mosquito species. mSphere.

[187] Merdić E, Lovaković T (2001). Population dynamic of *Aedes vexans* and *Ochlerotatus sticticus* in flooded areas of the River Drava in Osijek, Croatia. J Am Mosq Control Assoc.

[188] Merdić E, Krčmar S, Sudarić Bogojević M, Jeličić Ž (2007). Response of mosquitoes to different synthetic and natural olfactory attractants (Diptera, Culicidae). Entomol Generalis.

[189] Bogojević Sudarić M, Merdić E, Vrućina I, Merdić S, Zahirović Ž, Turić N (2008). Results of ten years of mosquito (Diptera: Culicidae) monitoring in Osijek, Croatia. Entomol Croat.

[190] Merdić E, Boca I, Bogojević MS, Landeka N (2008). Mosquitoes of Istria, a contribution to the knowledge of Croatian mosquito fauna (Diptera, Culicidae). Period Biol.

[191] Bogojević Sudarić M, Merdić E, Turić N, Jeličić Ž, Zahirović Ž, Vrućina I (2009). Seasonal dynamics of mosquitoes (Diptera: Culicidae) in Osijek (Croatia) for the period 1995–2004. Biologia (Bratisl).

[192] Merdić E, Bogojević MS, Boca I, Turić N (2010). Determined and estimated mosquito (Diptera, Culicidae) fauna in the city of Osijek, Croatia, using dry-ice baited CDC traps. Period Biol.

[193] Merdić E (2013). Mosquitoes—vectors of West Nile virus in Croatia. Rad Croat Acad Sci Arts Med Sci.

[194] Rettich F, Imrichova K, Šebesta O (2007). Seasonal comparisons of the mosquito fauna in the flood plains of Bohemia and Moravia, Czech Republic. Eur Mosq Bull.

[195] Votýpka J, Šeblová V, Rádrová J (2008). Spread of the West Nile virus vector *Culex modestus* and the potential malaria vector *Anopheles hyrcanus* in central Europe. J Vector Ecol.

[196] Šebesta O, Rettich F, Minar J, Halouzka J, Hubalek Z, Juricova Z (2009). Presence of the mosquito *Anopheles hyrcanus* in South Moravia, Czech Republic. Med Vet Entomol.

[197] Dvořák L (2012). *Culiseta glaphyroptera* (Schiner, 1864): a common species in the southwestern Czech Republic. Eur Mosq Bull.

[198] Hubalek Z, Sebesta O, Pesko J, Betasova L, Blazejova H, Venclikova K (2014). Isolation of Tahyna virus (California Encephalitis Group) from *Anopheles hyrcanus* (Diptera, Culicidae), a mosquito species new to, and expanding in, Central Europe. J Med Entomol.

[199] Šebesta O, Gelbič I (2015). Increased presence of the thermophilic mosquitoes and potential vectors *Anopheles hyrcanus* (Pallas 1771) and *Culex modestus* Ficalbi 1889 in Central Europe’s lower Dyje River basin (South Moravia, Czech Republic). Ann Soc Entomol Fr.

[200] Šebesta O, Gelbič I (2016). Late flooding combined with warm autumn—potential possibility for prolongation of transmission of mosquito-borne diseases. Biologia (Bratisl).

[201] Rudolf I, Betášová L, Blažejová H, Venclíková K, Straková P, Šebesta O (2017). West Nile virus in overwintering mosquitoes, central Europe. Parasites Vectors.

[202] Rudolf I, Šikutová S, Šebesta O, Mendel J, Malenovský I, Kampen H (2020). Overwintering of *Culex modestus* and other mosquito species in a reedbed ecosystem, including arbovirus findings. J Am Mosq Control Assoc.

[203] Miaoulis M, Giantsis IA, Schaffner F, Chaskopoulou A (2018). Re-examination of the taxonomic status of *Anopheles hyrcanus* and *An. pseudopictus* using a multilocus genetic approach. J Vector Ecol.

[204] Herm R, Kirik H, Vilem A, Zani L, Forth JH, Müller A (2021). No evidence for African swine fever virus DNA in haematophagous arthropods collected at wild boar baiting sites in Estonia. Transbound Emerg Dis.

[205] Pradel JA, Martin T, Rey D, Foussadier R, Bicout DJ (2009). Is *Culex modestus* (Diptera: Culicidae), vector of West Nile virus, spreading in the Dombes Area, France?. J Med Entomol.

[206] Cook S, Chung BYW, Bass D, Moureau G, Tang S, McAlister E (2013). Novel virus discovery and genome reconstruction from field RNA samples reveals highly divergent viruses in dipteran hosts. PLoS ONE.

[207] Nebbak A, Koumare S, Illcox AC, Berenger J-M, Raoult D, Almeras L (2018). Field application of MALDI-TOF MS on mosquito larvae identification. Parasitology.

[208] Zoller T, Naucke TJ, May J, Hoffmeister B, Flick H, Williams CJ (2009). Malaria transmission in non-endemic areas: case report, review of the literature and implications for public health management. Malar J.

[209] Jost H, Bialonski A, Storch V, Gunther S, Becker N, Schmidt-Chanasit J (2010). Isolation and phylogenetic analysis of sindbis viruses from mosquitoes in Germany. J Clin Microbiol.

[210] Jöst H, Bialonski A, Schmetz C, Günther S, Becker N, Schmidt-Chanasit J (2011). Isolation and phylogenetic analysis of Batai virus, Germany. Am J Trop Med Hyg.

[211] Czajka C, Becker N, Poppert S, Jöst H, Schmidt-Chanasit J, Krüger A (2012). Molecular detection of *Setaria tundra* (Nematoda: Filarioidea) and an unidentified filarial species in mosquitoes in Germany. Parasit Vectors.

[212] Krüger A, Tannich E (2013). Rediscovery of *Anopheles algeriensis* Theob. (Diptera: Culicidae) in Germany after half a century. J Eur Mosq Control Assoc.

[213] Krüger A, Börstler J, Badusche M, Lühken R, Garms R, Tannich E (2014). Mosquitoes (Diptera: Culicidae) of metropolitan Hamburg, Germany. Parasitol Res.

[214] Börstler J, Jöst H, Garms R, Krüger A, Tannich E, Becker N (2016). Host-feeding patterns of mosquito species in Germany. Parasit Vectors.

[215] Pfitzner WP, Lehner A, Hoffmann D, Czajka C, Becker N (2018). First record and morphological characterization of an established population of *Aedes* (*Hulecoeteomyia*) *koreicus* (Diptera: Culicidae) in Germany. Parasit Vectors.

[216] Scheuch D, Schäfer M, Eiden M, Heym E, Ziegler U, Walther D (2018). Detection of Usutu, Sindbis, and Batai viruses in mosquitoes (Diptera: Culicidae) collected in Germany, 2011–2016. Viruses.

[217] Tippelt L, Walther D, Scheuch DE, Schäfer M, Kampen H (2018). Further reports of *Anopheles algeriensis* Theobald, 1903 (Diptera: Culicidae) in Germany, with evidence of local mass development. Parasitol Res.

[218] Heym EC, Kampen H, Krone O, Schäfer M, Werner D (2019). Molecular detection of vector-borne pathogens from mosquitoes collected in two zoological gardens in Germany. Parasitol Res.

[219] Pernat N, Kampen H, Jeschke JM, Werner D (2021). Buzzing homes: using citizen science data to explore the effects of urbanization on indoor mosquito communities. Insects.

[220] Linton Y-M, Smith L, Harbach RE (2002). Observations on the taxonomic status of *Anopheles subalpinus* Hackett & Lewis and *An. melanoon* Hackett. J Eur Mosq Control Assoc.

[221] Pastoula E, Samanidou-Voyadjoglou A, Spanakos G, Kremastinou J, Nasioulas G, Vakalis NC (2007). Molecular characterization of the *Anopheles maculipennis* complex during surveillance for the 2004 olympic games in Athens. Med Vet Entomol.

[222] Chaskopoulou A, Latham MD, Pereira RM, Connelly R, Bonds JA, Koehler PG (2011). Efficacy of aerial ultra-low volume applications of two novel water-based formulations of unsynergized pyrethroids against riceland mosquitoes in Greece. J Am Mosq Control Assoc.

[223] Akiner MM, Caglar SS, Simsek FM (2013). Yearly changes of insecticide susceptibility and possible insecticide resistance mechanisms of *Anopheles maculipennis* Meigen (Diptera: Culicidae) in Turkey. Acta Trop.

[224] Lytra I, Emmanouel N (2014). Study of *Culex tritaeniorhynchus* and species composition of mosquitoes in a rice field in Greece. Acta Trop.

[225] Beleri S, Chatzinikolaou S, Nearchou A, Patsoula E (2017). Entomological study of the mosquito fauna in the regional unit of Drama, region of East Macedonia-Thrace, Greece (2015 to 2016). Vector-Borne Zoonotic Dis.

[226] Fotakis EA, Chaskopoulou A, Grigoraki L, Tsiamantas A, Kounadi S, Georgiou L (2017). Analysis of population structure and insecticide resistance in mosquitoes of the genus *Culex*, *Anopheles* and *Aedes* from different environments of Greece with a history of mosquito borne disease transmission. Acta Trop.

[227] Pergantas P, Tsatsaris A, Malesios C, Kriparakou G, Demiris N, Tselentis Y (2017). A spatial predictive model for malaria resurgence in central Greece integrating entomological, environmental and social data. PLoS ONE.

[228] Karypidou MC, Almpanidou V, Tompkins AM, Mazaris AD, Gewehr S, Mourelatos S (2020). Projected shifts in the distribution of malaria vectors due to climate change. Clim Change.

[229] Fotakis EA, Giantsis IA, Castells Sierra J, Tanti F, Balaska S, Mavridis K (2020). Population dynamics, pathogen detection and insecticide resistance of mosquito and sand fly in refugee camps, Greece. Infect Dis Poverty.

[230] Patsoula E, Beleri S, Tegos N, Mkrtsian R, Vakali A, Pervanidou D (2020). Entomological data and detection of West Nile virus in mosquitoes in Greece (2014–2016), before disease re-emergence in 2017. Vector-Borne Zoonotic Dis.

[231] Spanoudis CG, Pappas CS, Savopoulou-Soultani M, Andreadis SS (2021). Composition, seasonal abundance, and public health importance of mosquito species in the regional unit of Thessaloniki, Northern Greece. Parasitol Res.

[232] Szentpáli-Gavallér K, Antal L, Tóth M, Kemenesi G, Soltész Z, Dán A (2014). Monitoring of West Nile virus in mosquitoes between 2011–2012 in Hungary. Vector-Borne Zoonotic Dis.

[233] Kemenesi G, Kurucz K, Kepner A, Dallos B, Oldal M, Herczeg R (2015). Circulation of *Dirofilaria repens*, *Setaria tundra*, and *Onchocercidae* species in Hungary during the period 2011–2013. Vet Parasitol.

[234] Sáringer-Kenyeres M, Kenyeres Z, Földvári G, Majoros G (2017). First record of mermithid larva (Nematoda: Mermithidae) in *Anopheles maculipennis* complex (Diptera: Culicidae) imago in Central-Europe. Biologia.

[235] Ashe P, O’Connor JP, Casey RJ (1991). Irish mosquitoes (Diptera: Culicidae): a checklist of the species and their known distribution. R Irish Acad.

[236] Ascoli V, Facchinelli L, Valerio L, Zucchetto A, Dal Maso L, Coluzzi M (2006). Distribution of mosquito species in areas with high and low incidence of classic Kaposi’s sarcoma and seroprevalence for HHV-8. Med Vet Entomol.

[237] Cancrini G, Magi M, Gabrielli S, Arispici M, Tolari F, Dell’Omodarme M (2006). Natural vectors of dirofilariasis in rural and urban areas of the Tuscan region, central Italy. J Med Entomol.

[238] Toma L, Cipriani M, Goffredo M, Romi R, Lelli R. First report on entomological field activities for the surveillance of West Nile disease in Italy. Vet Ital. 2008;44(3):483–97, 499–12. 20405446

[239] Ascoli V, Senis G, Zucchetto A, Valerio L, Facchinelli L, Budroni M (2009). Distribution of ‘promoter’ sandflies associated with incidence of classic Kaposi’s sarcoma. Med Vet Entomol.

[240] Calzolari M, Bonilauri P, Bellini R, Caimi M, Defilippo F, Maioli G (2010). Arboviral survey of mosquitoes in two northern italian regions in 2007 and 2008. Vector-Borne Zoonotic Dis.

[241] Talbalaghi A, Moutailler S, Vazeille M, Failloux A-B (2010). Are *Aedes albopictus* or other mosquito species from northern Italy competent to sustain new arboviral outbreaks?. Med Vet Entomol.

[242] Calzolari M, Bonilauri P, Bellini R, Albieri A, Defilippo F, Tamba M (2013). Usutu virus persistence and West Nile virus inactivity in the Emilia-Romagna Region (Italy) in 2011. PLoS ONE.

[243] Huhtamo E, Lambert AJ, Costantino S, Servino L, Krizmancic L, Boldorini R (2013). Isolation and full genomic characterization of Batai virus from mosquitoes, Italy 2009. J Gen Virol.

[244] Pautasso A, Desiato R, Bertolini S, Vitale N, Radaelli MC, Mancini M (2013). Mosquito surveillance in Northwestern Italy to monitor the occurrence of tropical vector-borne diseases. Transbound Emerg Dis.

[245] Flacio E, Rossi-Pedruzzi A, Bernasconi-casati E, Patocchi N (2014). Culicidae fauna from canton ticino and report of three new species for Switzerland. Mitt Schweiz Entomol Ges.

[246] Montarsi F, Mazzon L, Cazzin S, Ciocchetta S, Capelli G (2015). Seasonal and daily activity patterns of mosquito (Diptera: Culicidae) vectors of pathogens in Northeastern Italy. J Med Entomol.

[247] Llopis VI, Tomassone L, Grego E, Serrano E, Mosca A, Vaschetti G (2016). Evaluating the feeding preferences of West Nile virus mosquito vectors using bird-baited traps. Parasit Vectors.

[248] Verna F, Modesto P, Radaelli MC, Francese DR, Monaci E, Desiato R (2017). Control of mosquito-borne diseases in Northwestern Italy: preparedness from one season to the next. Vector-Borne Zoonotic Dis.

[249] Toma L, Catalani M, Catalano A, Goffredo M, Romi R, Di Luca M (2017). Finding of *Anopheles* (*Anopheles*) *hyrcanus* (Pallas, 1771) (Diptera, Culicidae) during the entomological surveillance for West Nile virus in Umbria, Italy. Vet Ital.

[250] Macaluso G, Gucciardi F, Guercio A, Blanda V, La Russa F, Torina A (2021). First neuroinvasive human case of West Nile disease in southern Italy: results of the ‘One Health’ approach. Vet Med Sci.

[251] Muja-Bajraktari N, Zhushi-Etemi F, Dikolli-Velo E, Kadriaj P, Gunay F (2019). The composition, diversity, and distribution of mosquito fauna (Diptera: Culicidae) in Kosovo. J Vector Ecol.

[252] Bernotiene R (2012). The fauna and seasonal activity of mosquitoes (Diptera: Culicidae) in the Curonian Spit (Russia, Lithuania). J Eur Mosq Control Assoc.

[253] Beck M, Galm M, Weitzel T, Fohlmeister V, Kaiser A, Amold A (2003). Preliminary studies on the mosquito fauna of Luxembourg. Eur Mosq Bull.

[254] Sulesco TM, Toderas IK, Toderas LG (2013). Annotated checklist of the mosquitoes of the Republic of Moldova. J Am Mosq Control Assoc.

[255] Sulesco TM, von Thien H, Toderas L, Lühken R, Tannich E (2016). Circulation of *Dirofilaria repens* and *Dirofilaria immitis* in Moldova. Parasit Vectors.

[256] Failloux AB, Bouattour A, Faraj C, Gunay F, Haddad N, Harrat Z (2017). surveillance of arthropod-borne viruses and their vectors in the Mediterranean and Black Sea regions within the MediLabSecure Network. Curr Trop Med Rep.

[257] Reusken C, De Vires VA, Ceelen E, Beeuwkes J, Scholte E-J (2011). A study of the circulation of West Nile virus, Sindbis virus, Batai virus and Usutu virus in mosquitoes in a potential high-risk area for arbovirus circulation in the Netherlands, “De Oostvaardersplassen”. J Eur Mosq Control Assoc.

[258] Scholte E-J, den Hartog W, Reusken C (2011). A report of *Anopheles algeriensis* (Diptera: Culicidae) from The Netherlands. Entomol Ber.

[259] Rydzanicz K, Lonc E (2003). Species composition and seasonal dynamics of mosquito larvae in the Wrocław, Poland area. J Vector Ecol.

[260] Wegner E (2007). Additions to the mosquito fauna (Diptera: Culicidae) of Wrocław, Poland. J Eur Mosq Control Assoc.

[261] Wegner E (2009). A study of mosquito fauna (Diptera: Culicidae) and the phenology of the species recorded in Wilanów (Warsaw, Poland). J Eur Mosq Control Assoc.

[262] Gliniewicz A, Rydzanicz K, Mikulak E (2015). Methods of mosquito plague control in Świnoujscie area based on the analysis of species distribution. Przegl Epidemiol.

[263] Rydzanicz K, Czułowska A, Dyczko D, Kiewra D (2021). Assessment of mosquito larvae (Diptera: Culicidae) productivity in urban cemeteries in Wroclaw (SW Poland). Int J Trop Insect Sci.

[264] Lopes P, Lourenco P, Sousa C, Novo T, Rodrigues A, Almeida PG, et al. Modelling patterns of mosquito density based on remote sensing images. Estoril Congress Center; 2005. p. 251–8. http://www.earsel.org/symposia/2005-symposium-Porto/pdf/031.pdf. Accessed 30 Nov 2020.

[265] Freitas FB, Novo MT, Esteves A, de Almeida APG (2012). Species composition and WNV screening of mosquitoes from Lagoons in a Wetland Area of the Algarve, Portugal. Front Physiol.

[266] Osório HC, Zé-Zé L, Alves MJ (2012). Host-feeding patterns of *Culex pipiens* and other potential mosquito vectors (Diptera: Culicidae) of West Nile virus (Flaviviridae) collected in Portugal. J Med Entomol.

[267] Benali A, Nunes JP, Freitas FB, Sousa CA, Novo MT, Lourenço PM (2014). Satellite-derived estimation of environmental suitability for malaria vector development in Portugal. Remote Sens Environ.

[268] Osório H, Zé-Zé L, Amaro F, Alves M (2014). Mosquito surveillance for prevention and control of emerging mosquito-borne diseases in Portugal—2008–2014. Int J Environ Res Public Health.

[269] Ferreira C, de Pinho MV, Novo M, Calado M, Gonçalves L, Belo S (2015). First molecular identification of mosquito vectors of *Dirofilaria immitis* in continental Portugal. Parasites Vectors.

[270] De Pinho MV, Mendes AM, Mauricio IL, Calado MM, Novo MT, Belo S (2016). Molecular detection of *Wolbachia pipientis* in natural populations of mosquito vectors of *Dirofilaria immitis* from continental Portugal: first detection in *Culex theileri*. Med Vet Entomol.

[271] Madeira S, Duarte A, Boinas F, Costa OH (2021). A DNA barcode reference library of Portuguese mosquitoes. Zoonoses Public Health.

[272] Török E, Tomazatos A, Cadar D, Horváth C, Keresztes L, Jansen S (2016). Pilot longitudinal mosquito surveillance study in the Danube Delta Biosphere Reserve and the first reports of *Anopheles algeriensis* Theobald, 1903 and *Aedes hungaricus* Mihályi, 1955 for Romania. Parasites Vectors.

[273] Ionică AM, Zittra C, Wimmer V, Leitner N, Votýpka J, Modrý D (2017). Mosquitoes in the Danube Delta: searching for vectors of filarioid helminths and avian malaria. Parasit Vectors.

[274] Tomazatos A, Cadar D, Török E, Maranda I, Horváth C, Keresztes L (2018). Circulation of *Dirofilaria immitis* and *Dirofilaria repens* in the Danube Delta Biosphere Reserve, Romania. Parasites Vectors.

[275] Aibulatov SV (2009). Bloodsucking dipterans (Diptera: Ceratopogonidae, Culicidae, Simuliidae, Tabanidae) of the Kurgala Peninsula, Leningrad Province. Entomol Rev.

[276] Grushko OG, Sharakhova MV, Stegnii VN, Sharakhov IV (2009). Molecular organization of heterochromatin in malaria mosquitoes of the *Anopheles maculipennis* subgroup. Gene.

[277] Lapshin DN, Vorontsov DD (2013). Frequency tuning of individual auditory receptors in female mosquitoes (Diptera, Culicidae). J Insect Physiol.

[278] Artemov G, Bondarenko S, Sapunov G, Stegniy V (2015). Tissue-specific differences in the spatial interposition of x-chromosome and 3r chromosome regions in the malaria mosquito *Anopheles messeae* Fall. PLoS ONE.

[279] Gordeev MI, Moskaev AV (2016). Chromosomal polymorphism in the populations of malaria mosquito *Anopheles messeae* (Diptera, Culicidae) in the Volga region. Russ J Genet.

[280] Nekrasova LS, Vigorov YL, Vigorov AY (2016). Dynamics of the composition of the fauna of mosquitoes (Diptera, Culicidae) in parks of Yekaterinburg. Russ J Ecol.

[281] Perevozkin VP, Bondarchuk SS, Kormilitsin AV (2018). Cytogenetic analysis of malarial mosquitoes of Kaliningrad Oblast. Russ J Genet.

[282] Shaikevich E, Bogacheva A, Ganushkina L (2019). *Dirofilaria* and *Wolbachia* in mosquitoes (Diptera: Culicidae) in central European Russia and on the Black Sea coast. Parasite.

[283] Shaikevich E, Bogacheva A, Rakova V, Ganushkina L, Ilinsky Y (2019). *Wolbachia* symbionts in mosquitoes: intra- and intersupergroup recombinations, horizontal transmission and evolution. Mol Phylogenet Evol.

[284] Vujić A, Stefanović A, Dragičević I, Matijević T, Pejčić L, Knežević M (2010). Species composition and seasonal dynamics of mosquitoes (Diptera: Culicidae) in flooded areas of Vojvodina, Serbia. Arch Biol Sci.

[285] Petric D, Cvjetkovic IH, Radovanov J, Cvjetkovic D, Patic VJ, Milosevic V (2012). West nile virus surveillance in humans and mosquitoes and detection of cell fusing agent virus in Vojvodina province (Serbia). HealthMED.

[286] Kemenesi G, Krtinić B, Milankov V, Kutas A, Dallos B, Oldal M (2014). West Nile virus surveillance in mosquitoes, April to October 2013, Vojvodina province, Serbia: implications for the 2014 season. Eurosurveillance.

[287] Petrić D, Petrović T, Hrnjaković Cvjetković I, Zgomba M, Milošević V, Lazić G (2017). West Nile virus ‘circulation’ in Vojvodina, Serbia: mosquito, bird, horse and human surveillance. Mol Cell Probes.

[288] Brestovský J, Jalili N (2001). Mosquitoes of the Ipeľ River Floodplain in the surroundings of the Šahy town after the floods in 1999. Acta Zool.

[289] Jalili N, Halgoš J (2004). Mosquito prevalence in the Komárno and Nové Zámky regions of southern Slovakia. J Eur Mosq Control Assoc.

[290] Strelková L, Halgoš J (2012). Mosquitoes (Diptera, Culicidae) of the Morava River floodplain, Slovakia. Open Life Sci.

[291] Bocková E, Kočišová A, Hlavatá H (2013). Evaluation of species composition and seasonal dynamics of mosquito larvae in the Košice Basin during 2010 and 2011. Biologia.

[292] Bocková E, Iglódyová A, Kočišová A (2015). Potential mosquito (Diptera:Culicidae) vector of *Dirofilaria repens* and *Dirofilaria immitis* in urban areas of Eastern Slovakia. Parasitol Res.

[293] Bocková E, Kočišová A (2016). Species composition of mosquitoes (Diptera: Culicidae) in relation to climate conditions in South-Eastern Slovakia. Biologia (Bratisl).

[294] Bargues MD, Morchón R, Latorre JM, Cancrini G, Mas-Coma S, Simón F (2006). Ribosomal DNA second internal transcribed spacer sequence studies of Culicid vectors from an endemic area of *Dirofilaria immitis* in Spain. Parasitol Res.

[295] Bargues MD, Latorre JM, Morchon R, Simon F, Escosa R, Aranda C (2006). rDNA sequences of *Anopheles* species from the Iberian peninsula and an evaluation of the 18S rRNA gene as phylogenetic marker in anophelinae. J Med Entomol.

[296] Aranda C, Sánchez-Seco MP, Cáceres F, Escosa R, Gálvez JC, Masià M (2009). Detection and monitoring of mosquito flaviviruses in Spain between 2001 and 2005. Vector-Borne Zoonotic Dis.

[297] Bueno-Marí R, Jiménez-Peydró R (2010). New anopheline records from the Valencian Autonomous Region of Eastern Spain (Diptera: Culicidae: Anophelinae). J Eur Mosq Control Assoc.

[298] Alba A, Allepuz A, Napp S, Soler M, Selga I, Aranda C (2012). Ecological surveillance for West Nile in Catalonia (Spain), learning from a five-year period of follow-up. Zoonoses Public Health.

[299] Bueno-Marí R, Bernués Bañeres A, Chordá-Olmos FA, Jiménez-Peydró R (2012). Entomological surveillance in a recent autochthonous malaria area of Spain. J Vector-Borne Dis.

[300] Bernués-Bañares A, Jiménez- PR (2013). Diversity of mosquitoes (Diptera Culicidae) in protected natural parks from Valencian Autonomous Region (Eastern Spain). Biodivers J.

[301] Roiz D, Ruiz S, Soriguer R, Figuerola J (2014). Climatic effects on mosquito abundance in Mediterranean wetlands. Parasit Vectors.

[302] Martínez-de la Puente J, Méndez M, Ruiz S, Godoy JA, Soriguer RC, Figuerola J (2015). Individual identification of endangered species using mosquito blood meals: a proof-of-concept study in Iberian lynx. Parasitol Res.

[303] Bravo-Barriga D, Gomes B, Almeida APG, Serrano-Aguilera FJ, Pérez-Martín JE, Calero-Bernal R (2017). The mosquito fauna of the western region of Spain with emphasis on ecological factors and the characterization of *Culex pipiens* forms. J Vector Ecol.

[304] Martínez-de la Puente J, Ferraguti M, Ruiz S, Roiz D, Llorente F, Pérez-Ramírez E (2018). Mosquito community influences West Nile virus seroprevalence in wild birds: implications for the risk of spillover into human populations. Sci Rep.

[305] Ruiz-Arrondo I, McMahon BJ, Hernández-Triana LM, Santibañez P, Portillo A, Oteo JA (2019). Surveillance of mosquitoes (Diptera, Culicidae) in a Northern Central Region of Spain: implications for the medical community. Front Vet Sci.

[306] González MA, Prosser SW, Hernández-Triana LM, Alarcón-Elbal PM, Goiri F, López S (2020). Avian feeding preferences of *Culex pipiens* and *Culiseta* spp. along an urban-to-wild gradient in northern Spain. Front Ecol Evol.

[307] Blackmore MS, Dahl C (2002). Field evaluation of a new surveillance trap in Sweden. J Am Mosq Control Assoc.

[308] Schäfer ML, Lundström JO, Petersson E (2008). Comparison of mosquito (Diptera: Culicidae) populations by wetland type and year in the lower river Dalälven region, Central Sweden. J Vector Ecol.

[309] Lundström J, Schäfer M, Hesson J, Blomgren E, Lindström A, Wahlqvist P (2013). The geographic distribution of mosquito species in Sweden. J Eur Mosq Control Assoc.

[310] Schäfer ML, Wahlqvist P, Lundström JO (2018). The Nedre Dalälven River Landscape in Central Sweden—a hot-spot for mosquito (Diptera: Culicidae) diversity. J Eur Mosq Control Assoc.

[311] Briegel H, Kaeslin M, Proft J (2002). Anopheles maculipennis complex in Switzerland: reassessing taxonomic status and malaria potential. Mitt Schweiz Entomol Ges.

[312] Yang T-C, Casati S, Flacio E, Caminada AP, Ruggeri-Bernardi N, Demarta A (2010). Detection of Chikungunya virus and arboviruses in mosquito vectors. J Entomol Sci.

[313] Schaffner F, Mathis A. Spatio-temporal diversity of the mosquito fauna (Diptera: Culicidae) in Switzerland. Final report. Ittigen: Swiss Federal Office for the Environment; 2013.

[314] Schoenenberger AC, Wagner S, Tuten HC, Schaffner F, Torgerson P, Furrer S (2016). Host preferences in host-seeking and blood-fed mosquitoes in Switzerland. Med Vet Entomol.

[315] Wagner S, Guidi V, Torgerson PR, Mathis A, Schaffner F (2018). Diversity and seasonal abundances of mosquitoes at potential arboviral transmission sites in two different climate zones in Switzerland. Med Vet Entomol.

[316] Lüleyap HU, Alptekin D, Kasap H, Kasap M (2002). Detection of knockdown resistance mutations in *Anopheles sacharovi* (Diptera: Culicidae) and genetic distance with *Anopheles gambiae* (Diptera: Culicidae) using cDNA sequencing of the voltage-gated sodium channel gene. J Med Entomol.

[317] Yurttas H, Alten B, Aytekin AM (2005). Variability in natural populations of *Anopheles sacharovi* (Diptera: Culicidae) from southeast Anatolia, revealed by morphometric and allozymic analyses. J Vector Ecol.

[318] Aldemir A, Demirci B, Kirpik MA, Alten B, Baysal A (2009). Species composition and seasonal dynamics of Mosquito larvae (Diptera: Culicidae) in Iǧdır plain, Turkey. Kafkas Univ Vet Fak Derg.

[319] Aldemir A (2009). Initial and residual activity of VectoBac 12 AS, VectoBac WDG, and VectoLex WDG for control of mosquitoes in Ararat Valley, Turkey. J Am Mosq Control Assoc.

[320] Aytekin S, Aytekin AM, Alten B (2009). Effect of different larval rearing temperatures on the productivity (R o) and morphology of the malaria vector *Anopheles superpictus* Grassi (Diptera: Culicidae) using geometric morphometrics. J Vector Ecol.

[321] Şimşek F, Kaynaş S, Toz S, Özbel Y, Alten B, Chan AS (2010). Evaluation of the VecTestTM malaria antigen panel assay using *Anopheles sacharovi* specimens in an endemic area, Sanliurfa province, Turkey. Kafkas Universitesi Veteriner Fakultesi Dergisi.

[322] Yildirim A, Inci A, Duzlu O, Biskin Z, Ica A, Sahin I (2011). *Aedes vexans* and *Culex pipiens* as the potential vectors of *Dirofilaria immitis* in Central Turkey. Vet Parasitol.

[323] Inci A, Yildirim A, Njabo KY, Duzlu O, Biskin Z, Ciloglu A (2012). Detection and molecular characterization of avian Plasmodium from mosquitoes in central Turkey. Vet Parasitol.

[324] Ergunay K, Gunay F, Oter K, Kasap OE, Orsten S, Akkutay AZ (2013). Arboviral surveillance of field-collected mosquitoes reveals circulation of West Nile virus lineage 1 strains in Eastern Thrace, Turkey. Vector-Borne Zoonotic Dis.

[325] Ocal M, Orsten S, Inkaya AC, Yetim E, Acar NP, Alp S (2014). Ongoing activity of Toscana virus genotype A and West Nile virus lineage 1 strains in Turkey: a clinical and field survey. Zoonoses Public Health.

[326] Duzlu O, Yildirim A, Inci A, Gumussoy KS, Ciloglu A, Onder Z (2016). Molecular investigation of Francisella-like endosymbiont in ticks and *Francisella tularensis* in Ixodid ticks and mosquitoes in Turkey. Vector-Borne Zoonotic Dis.

[327] Demirci B, Bedir H, Taskin Tasci G, Vatansever Z (2021). Potential mosquito vectors of *Dirofilaria immitis* and *Dirofilaira repens* (Spirurida: Onchocercidae) in Aras Valley, Turkey. J Med Entomol.

[328] Gazzavi-Rogozina L, Filiptsova O, Naboka O, Ochkur A (2016). The species composition of malaria mosquitoes in the Kharkov Region (Ukraine): natural factors of malaria spread. Gazi Med J.

[329] Szanyi K, Nagi A, Molnàr A, Szabò LJ, Szani S (2020). Mosquito (Diptera: Culicidae) fauna of the Velyka Dobron’ game reserve (West Ukraine) with new distribution data and medical risk assessment. Turk J Zool.

[330] Ayres C, Müller P, Dyer N, Wilding C, Rigden D, Donnelly M (2012). Correction: comparative genomics of the anopheline glutathione *s*-transferase epsilon cluster. PLoS ONE.

[331] Golding N, Nunn MA, Medlock JM, Purse BV, Vaux AG, Schäfer SM (2012). West Nile virus vector *Culex modestus* established in southern England. Parasit Vectors.

[332] Medlock JM, Vaux AGC (2015). Seasonal dynamics and habitat specificity of mosquitoes in an English wetland: implications for UK wetland management and restoration. J Vector Ecol.

[333] Quintavalle Pastorino G, Albertini M, Carlsen F, Cunningham AA, Daniel BA, Flach E (2015). Project MOSI: rationale and pilot-study results of an initiative to help protect zoo animals from mosquito-transmitted pathogens and contribute data on mosquito spatio-temporal distribution change. Int Zoo Yearb.

[334] Vaux AG, Gibson G, Hernandez-Triana LM, Cheke RA, McCracken F, Jeffries CL (2015). Enhanced West Nile virus surveillance in the North Kent marshes, UK. Parasit Vectors.

[335] Fernández de Marco M, Brugman VA, Hernández-Triana LM, Thorne L, Phipps LP, Nikolova NI (2016). Detection of *Theileria orientalis* in mosquito blood meals in the United Kingdom. Vet Parasitol.

[336] Hernandez-Colina A, Gonzalez-Olvera M, Lomax E, Townsend F, Maddox A, Hesson JC, et al. Blood-feeding ecology of mosquitoes in two zoological gardens in the United Kingdom. Parasit Vectors. 2021;14:249. 10.1186/s13071-021-04735-0. 10.1186/s13071-021-04735-0 PMC8139098 34016159

[337] Sousa CA. Malaria vectorial capacity and competence of *Anopheles atroparvus* Van Thiel, 1927 (Diptera, Culicidae): Implications for the potential re-emergence of malaria in Portugal. PhD thesis. Lisbon: Universidade Nova de Lisboa; 2008.

